# Kinesin-3 motors are fine-tuned at the molecular level to endow distinct mechanical outputs

**DOI:** 10.1186/s12915-022-01370-8

**Published:** 2022-08-10

**Authors:** Pushpanjali Soppina, Nishaben Patel, Dipeshwari J. Shewale, Ashim Rai, Sivaraj Sivaramakrishnan, Pradeep K. Naik, Virupakshi Soppina

**Affiliations:** 1 Discipline of Biological Engineering, Indian Institute of Technology Gandhinagar, Gandhinagar, Gujarat 382355 India; 2 Department of Biotechnology and Bioinformatics, Sambalpur University, Sambalpur, Orissa 768019 India; 3 Department of Genetics, Cell Biology and Development, University of Minnesota, Minnesota, MN 55455 USA

**Keywords:** Chemomechanical, Kinesin-3, Microtubule bending, Baculovirus, Superprocessive, ATPases

## Abstract

**Background:**

Kinesin-3 family motors drive diverse cellular processes and have significant clinical importance. The ATPase cycle is integral to the processive motility of kinesin motors to drive long-distance intracellular transport. Our previous work has demonstrated that kinesin-3 motors are fast and superprocessive with high microtubule affinity. However, chemomechanics of these motors remain poorly understood.

**Results:**

We purified kinesin-3 motors using the Sf9-baculovirus expression system and demonstrated that their motility properties are on par with the motors expressed in mammalian cells. Using biochemical analysis, we show for the first time that kinesin-3 motors exhibited high ATP turnover rates, which is 1.3- to threefold higher compared to the well-studied kinesin-1 motor. Remarkably, these ATPase rates correlate to their stepping rate, suggesting a tight coupling between chemical and mechanical cycles. Intriguingly, kinesin-3 velocities (KIF1A > KIF13A > KIF13B > KIF16B) show an inverse correlation with their microtubule-binding affinities (KIF1A < KIF13A < KIF13B < KIF16B). We demonstrate that this differential microtubule-binding affinity is largely contributed by the positively charged residues in loop8 of the kinesin-3 motor domain. Furthermore, microtubule gliding and cellular expression studies displayed significant microtubule bending that is influenced by the positively charged insert in the motor domain, K-loop, a hallmark of kinesin-3 family.

**Conclusions:**

Together, we propose that a fine balance between the rate of ATP hydrolysis and microtubule affinity endows kinesin-3 motors with distinct mechanical outputs. The K-loop, a positively charged insert in the loop12 of the kinesin-3 motor domain promotes microtubule bending, an interesting phenomenon often observed in cells, which requires further investigation to understand its cellular and physiological significance.

**Supplementary Information:**

The online version contains supplementary material available at 10.1186/s12915-022-01370-8.

## Background

Kinesin motors are ATPases that convert the chemical energy of adenosine triphosphate (ATP) into mechanical work, which enables their movement on microtubules (MTs) to carry out multiple cellular processes, such as intracellular transport and cell division [1–3]. Kinesins contain a highly conserved ~ 350 amino acid catalytic motor domain responsible for MT binding and ATP hydrolysis. Although the kinesin motor domains exhibit a high degree of sequence conservation (50% identity) between different subfamilies, their cellular functions have remarkable diversity [4]. Our current understanding of kinesins’ mechanical and biophysical properties is derived mainly from the studies on kinesin-1, the founding member of the kinesin superfamily. A dimeric (two-headed) kinesin motor undergoes alternative ATP hydrolysis successively to take 8-nm steps along the MT surface in a hand-over-hand manner to drive intracellular cargo transport [5–8]. The landing of ADP-bound motor on the MT triggers ADP release and ATP binding. This conformational change generates a power stroke to relocate the rear motor domain to the forward binding site along the MT track. Subsequently, ATP in the rear head is hydrolyzed, allowing it to detach from the MT [9].

Kinesin-3 is one of the largest families among the kinesin superfamily, consisting of five subfamilies (KIF1, KIF13, KIF14, KIF16, and KIF28) [10]. Kinesin-3 members are involved in numerous essential cellular processes, such as intracellular transport, endocytosis, signaling, and cell division. Defects in kinesin-3 can lead to various illnesses and malfunctions, including developmental defects, neurodegenerative disorders, and cancer [11–16]. Despite the high sequence conservation of motor domains, members of kinesin-3 motors have been demonstrated to possess distinct characteristics compared to other kinesin motors. Dimeric kinesin-3 motors are fast, are superprocessive, and have high MT affinity [17–20]. However, the ATP turnover cycle of these highly processive kinesins is poorly studied. The ATP turnover cycle is fundamental to the action of motor proteins along the MTs. Alteration in the ATP turnover cycle provides one way the characteristic motor domain can be tuned to diversity in the function observed for kinesin-3 motors. Studies on several kinesin and myosin motors have demonstrated a direct link between the rate at which ATP is hydrolyzed and the speed of cargo transport [4, 21]. Therefore, understanding kinesin-3 chemomechanical cycle is critical for interpreting the motor’s diverse cargo transport properties and cellular functions. Most importantly, how kinesin motors have evolutionarily adapted to accomplish functional diversity is of particular biological significance. Therefore, dissection of ATPase turnover cycle of a kinesin, both in the presence and absence of MTs, can help interpret the observed behavior.

In the present work, we establish purification of kinesin-3 motors using *Spodoptera frugiperda* (Sf9)-baculovirus expression system and show that their motility properties are identical to that of motors prepared from mammalian cell lysates. The MT-stimulated ATPase analysis from purified kinesin-3 motors exhibited significantly higher ATP turnover rates compared to kinesin-1 motor. The measured ATPase rates of kinesin-3 motors show tight coupling with their velocities measured in vitro, owing to which kinesin-3 motors take 8-nm steps [22, 23]. Intriguingly, the kinesin-3 motor velocities (KIF1A > KIF13A > KIF13B > KIF16B) show an inverse relationship with their MT-binding affinities (KIF1A < KIF13A < KIF13B < KIF16B). The positively charged residues in Loop8 largely influence the above MT affinities measured for kinesin-3 motors [24]. In the multi-motor gliding assay, unlike kinesin-1, family-specific insert in the kinesin-3 motor domain, the K-loop, besides influencing MT on-rate, also induces MT bending, a phenomenon commonly found inside the cells. However, its mechanism of regulation and in vivo importance requires further investigation. In conclusion, we demonstrate that kinesin-3 motors are evolutionarily fine-tuned at the molecular level to generate family-specific mechanical outputs, critical for cellular functions.

## Results

Kinesin-3 motors are built with unique mechanical outputs, such as high velocity, superprocessivity and a strong MT-binding affinity than other kinesin family motors [17–20]. A single kinesin-3 motor can take thousands of steps before detaching from the microtubule and is fueled by ATP hydrolysis [18]. Despite rigorous motility analysis over the last few years [22, 25–30], the basic chemomechanical properties of these motors remain poorly studied. Often such analysis requires purified, soluble, active motor proteins. The prokaryotic expression system is used widely for recombinant protein expression. As a control, we used a constitutively active version of kinesin-1, KHC(1–560), the founding member of the kinesin superfamily, whose motility properties are well characterized. The expression and purification of KHC(1–560) in bacteria resulted in multiple low molecular bands (Additional file 1: Fig. S1A, B) and protein degradation. Additionally, single-molecule motility analysis resulted in rare motility events and majority of them were bound non-specifically to the glass surface. Studies have shown that bacterial expression and purification of motor proteins usually result in a large population of dead motors due to improper protein folding and/or limited capacity of the host system [31–34]. Thus, an additional step of microtubule affinity purification is critical to eliminate dead and inactive motor fractions to some extent [35, 36].

To overcome these setbacks, we used baculovirus expression, one of the most powerful and versatile eukaryotic expression systems. Baculovirus has a strong polyhedrin promoter to drive the high-level expression of heterologous genes. In addition, this system employs the ability of cultured Sf9 cells to perform post-translational modification of expressed proteins, similar to those that occur in the natural host cell. Thus, we generated bacmids to purify full-length and constitutively active versions of kinesin-3 and kinesin-1 motors (Additional file 1: Fig. S2A-C) and subjected them to a one-step purification approach (Additional file 1: Fig. S3A-C). Size exclusion-high-performance liquid chromatography (SEC-HPLC) and circular dichroism (CD) analysis of these purified motor proteins showed a homogeneous protein population with regular secondary structures and protein folding, respectively. For representation, HPLC elution profile and CD spectra of KIF1A(1-393LZ)-mCit-FLAG are shown in Additional file 1: Fig. S3D, E. These Sf9-purified motors were used for detailed biochemical and biophysical characterizations.

### Sf9-purified kinesin-3 motors are fast and superprocessive

**Fig. 1:**
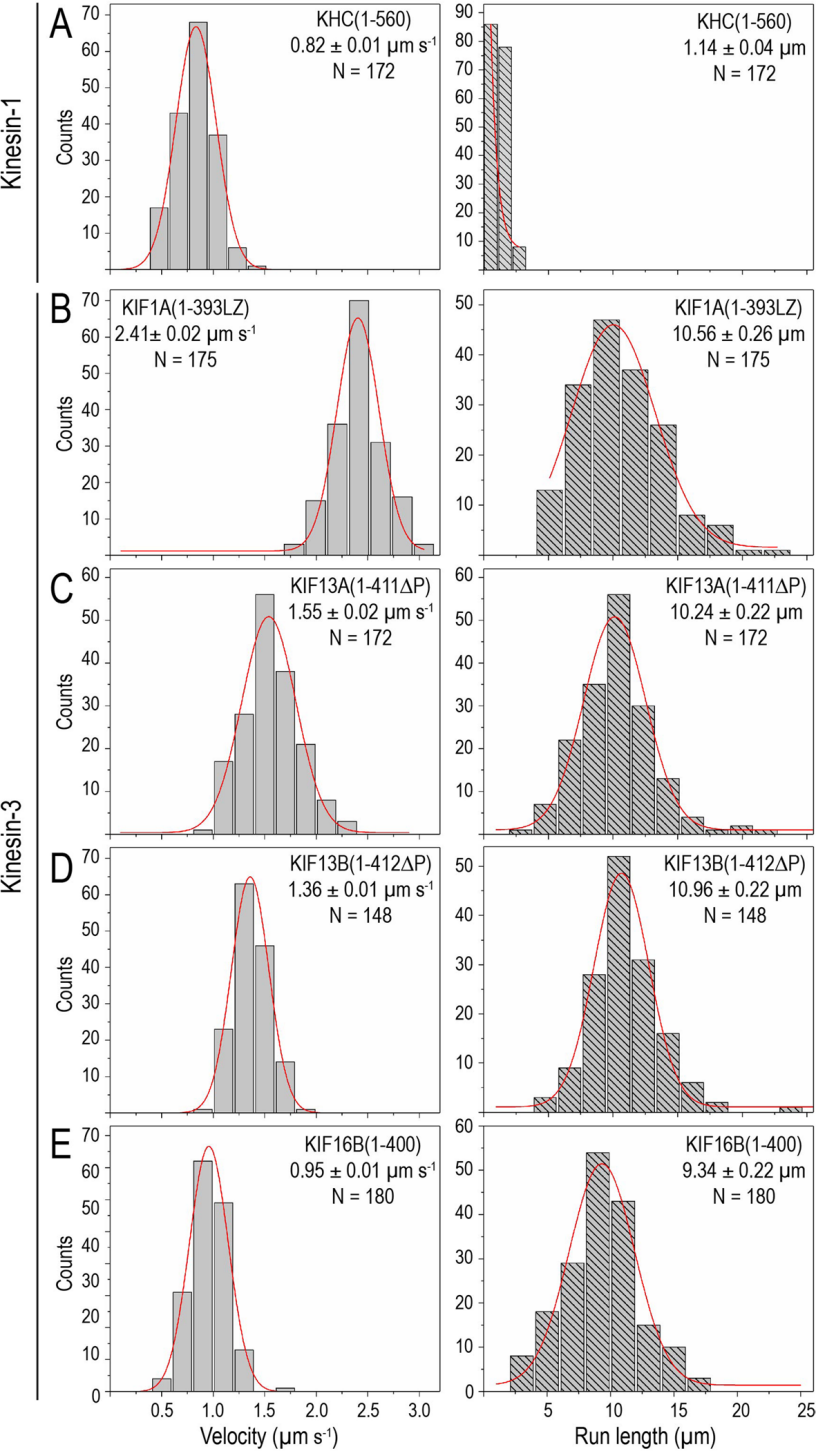
Sf9-baculovirus-purified kinesin-3 motors are robust and superprocessive. **A–E** Fluorescently tagged truncated constitutively active motors were purified using Sf9-baculovirus expression system. In vitro single-molecule motility assays of Sf9-purified **A** kinesin-1, KHC (1–560) and **B–E** kinesin-3 motor **B** KIF1A(1-393LZ), **C** KIF13A(1-411ΔP), **D** KIF13B(1-412ΔP), and **E** KIF16B(1–400) motors. For each population of motors, histograms of velocities (left panel) and run lengths (right panel) were plotted and fit to a single Gaussian. Average velocity and run length of the corresponding population of motors (N) are indicated on top-right or left corner as mean ± SEM. Data presented from three independent experiments

First, we wanted to establish whether Sf9-purified kinesin-3 motors can support MT-based superprocessive motility. We performed in vitro single-molecule motility assays of dimeric active kinesin-3 motors as described previously [17, 18]. The control constitutively active kinesin-1, KHC(1–560) motor, showed processive motion along the MT tracks with an average velocity of 0.82 µm s^−1^ and 1.14 ± 0.04 µm run length (Fig. 1A; Additional file 1: Fig. S4A; Table 1) [6, 7, 18, 37]. As kinesin-1 motor takes 8-nm steps [7, 8, 38], the measured velocity renders a stepping rate (average number of steps taken in 1 s) of 102.15 s^−1^ and a mean run time (average time spent on the MT) of 1.39 ± 0.3 s with a motor off-rate (frequency of motor detachment from the MT in 1 s) 0.72 ± 0.05 s^−1^.

**Table 1:** Biochemical and biophysical properties of kinesin-3 family motors

**Single-molecule motility assay**					
**Motor**	**Velocity (μm s**^**−1**^**)**	**Run length (μm)**	**Estimated stepping rate (s**^**−1**^**)**	**Mean run time (s)**	**Detachment rate (s**^**−1**^**)**
KIF1A(1-393LZ)	2.41 ± 0.02	10.56 ± 0.26	301.29	4.38 ± 1.3	0.23 ± 0.05
KIF13A(1-411ΔP)	1.55 ± 0.02	10.24 ± 0.22	194.19	6.61 ± 2.6	0.15 ± 0.07
KIF13B(1-412ΔP)	1.36 ± 0.01	10.96 ± 0.22	170.16	8.06 ± 2.4	0.12 ± 0.09
KIF16B(1–400)	0.95 ± 0.01	9.34 ± 0.22	118.75	9.83 ± 1.2	0.10 ± 0.02
KHC(1–560)	0.82 ± 0.01	1.14 ± 0.04	102.15	1.39 ± 0.3	0.72 ± 0.05
**ATPase assay**					
**Motor**	***k***_**cat**_** (s**^**−1**^**)**	***K***_**m**_** (µM)**	**Basal activity (s**^**−1**^**)**	**Step size (nm)**	
KIF1A(1-393LZ)	290.9 ± 8.69	7.03 ± 0.79	0.08	8.28	
KIF13A(1-411ΔP)	181.7 ± 8.31	5.16 ± 0.84	0.014	8.53	
KIF13B(1-412ΔP)	171.8 ± 7.82	4.23 ± 0.74	0.08	7.91	
KIF16B(1–400)	124.7 ± 6.94	2.43 ± 0.58	0.32	7.61	
KHC(1–560)	95.44 ± 15.5	10.23 ± 3.63	0.044	8.54	
**Microtubule gliding assay**					
**Motor**	**Gliding velocity (μm s**^**−1**^**)**				
KIF1A(1-393LZ)	1.16 ± 0.14				
KIF13A(1-411ΔP)	0.58 ± 0.05				
KIF13B(1-412ΔP)	0.60 ± 0.06				
KIF16B(1–400)	0.48 ± 0.08				
KHC(1–560)	0.46 ± 0.06				

Next, single-molecule motility analysis of Sf9-purified active dimeric kinesin-3 motors displayed robust superprocessive motion with high velocities along the MT (Fig. 1B–E; Additional file 1: Fig. S4B-E; Table 1) when compared to kinesin-1. It has been shown that the wild-type KIF1A(1–393) motors exhibit diffusive motion and short processive motion in vitro owing to weak neck coil dimerization potential [18, 28]. Therefore, we used its stable version, KIF1A(1-393LZ), in which a leucine zipper (LZ) segment of GCN4 was fused to the C-terminus of KIF1A(1–393) [18]. The KIF1A(1-393LZ) motor traveled with an average speed of 2.41 ± 0.02 µm s^−1^ and a distance of 10.56 ± 0.26 µm (Fig. 1B; Additional file 1: Fig. S4B; Table 1), akin to our previous measurements using mammalian cell lysates [17, 18]. As KIF1A motors take 8-nm step/ATP hydrolysis [22, 23, 28], the determined velocity corresponds to a stepping rate of 301.29 s^−1^, which is approximately threefold faster than kinesin-1. Furthermore, considering the determined average run length and the velocity over the total track, we computed a motor average run time of 4.38 ± 1.3 s, which corresponds to an off-rate of 0.23 ± 0.05 s^−1^.

The wild-type KIF13A(1–411) and KIF13B(1–412) motors exist as inactive monomers due to proline-mediated intramolecular neck coil-coiled coil1 (NC-CC1) interaction [18]. We, therefore, used proline-deleted dimeric versions, KIF13A(1-411ΔP390) and KIF13B(1-412ΔP391), respectively. The motility analysis of KIF13A(1-411ΔP390) [from now on referred to as KIF13A(1-411ΔP)] and KIF13B(1-412ΔP391) [from now on referred to as KIF13B(1-412ΔP)], exhibited average velocities of 1.55 ± 0.02 µm s^−1^ and 1.36 ± 0.01 µm s^−1^ and run lengths of 10.24 ± 0.22 µm and 10.96 ± 0.22 µm, respectively (Fig. 1C,D; Additional file 1: Fig. S4C-D; Table 1). Assuming these motors take 8-nm steps, their velocities reveal stepping rates of 194.19 s^−1^ and 170.16 s^−1^, respectively. Furthermore, based on the measured average run lengths and the velocities over the total track, we computed an average run time of 6.61 ± 2.6 s and 8.06 ± 2.4 s, which corresponds to motor detachment rates of 0.15 ± 0.07 s^−1^ and 0.12 ± 0.09 s^−1^, respectively.

Furthermore, motility analysis with active KIF16B(1–400) also exhibited long uniform motion along the MT with an average velocity of 0.95 ± 0.01 µm s^−1^ and run length 9.34 ± 0.22 µm (Fig. 1E; Additional file 1: Fig. S4E; Table 1). These motility properties showed remarkable consensus with previously measured kinesin-3 motility properties using mammalian cell lysate [17–19, 23, 39]. Considering KIF16B takes 8-nm steps, the determined velocity translates to a stepping rate of 118.75 s^−1^. Based on the measured run length and the velocity over the entire track, we found that the motor run time of 9.83 ± 1.2 s corresponds to a motor off-rate of 0.10 ± 0.02 s^−1^.

Together, these results demonstrate that the functional output of motor proteins purified from the Sf9-baculovirus system are comparable to those expressed in mammalian cells. Their motility properties are on par with those measured previously using mammalian cell lysates. The results also suggest that the high velocity and superprocessivity of kinesin-3 motors is inherent to their motor domains. Notably, this one-step motor purification protocol can be adapted to purify any other protein of interest.

### Kinesin-3 motors exhibit high ATP turnover rates

The ability of kinesin motors to take processive steps along the microtubule is tightly coupled to the ATP hydrolysis cycle because binding of ATP causes conformational changes in the motor domain. Studies on kinesin-1 and myosin have directly correlated their velocity and ATP turnover rate [4, 40]. However, previous chemomechanical studies of kinesin-3 motors have reported significantly lower ATP turnover rates [21, 25, 26, 28], which does not correlate with the measured high velocity and superprocessive motility [18, 19]. Thus, understanding the MT-stimulated chemomechanical behavior of kinesin-3 motors can help to elucidate the observed unique kinesin-3 motility properties. Therefore, we decided to do MT-stimulated ATPase measurements of full-length and constitutively active kinesin-3 motors. To do this, we adapted an ATPase assay based on phosphomolybdate complex formation, as described for smooth muscle myosin [41].

As a control, we used a dimeric KHC(1–560), constitutively active kinesin-1 motor whose catalytic rate constants have been well characterized, to optimize assay conditions. First, we determined the optimal motor concentration required to measure the ATPase activity by assaying a range of KHC(1–560) motor concentrations (1 to 100 nM) with a constant MT concentration. We found that a motor concentration of 10 nM is optimal for reliable and consistent measurement of ATPase activity across the preparations [24]. Next, we determined the range of MT concentrations desired to accurately measure ATPase activity for the individual motor type, depending on when the ATPase activity attains its steady-state maxima. For reliable ATPase measurements, an assay mixture containing purified motor protein, ATP, and varying MTs concentrations was incubated at room temperature for 2 h. Samples were collected every 30 min and inorganic phosphate release was measured [41].

**Fig. 2:**
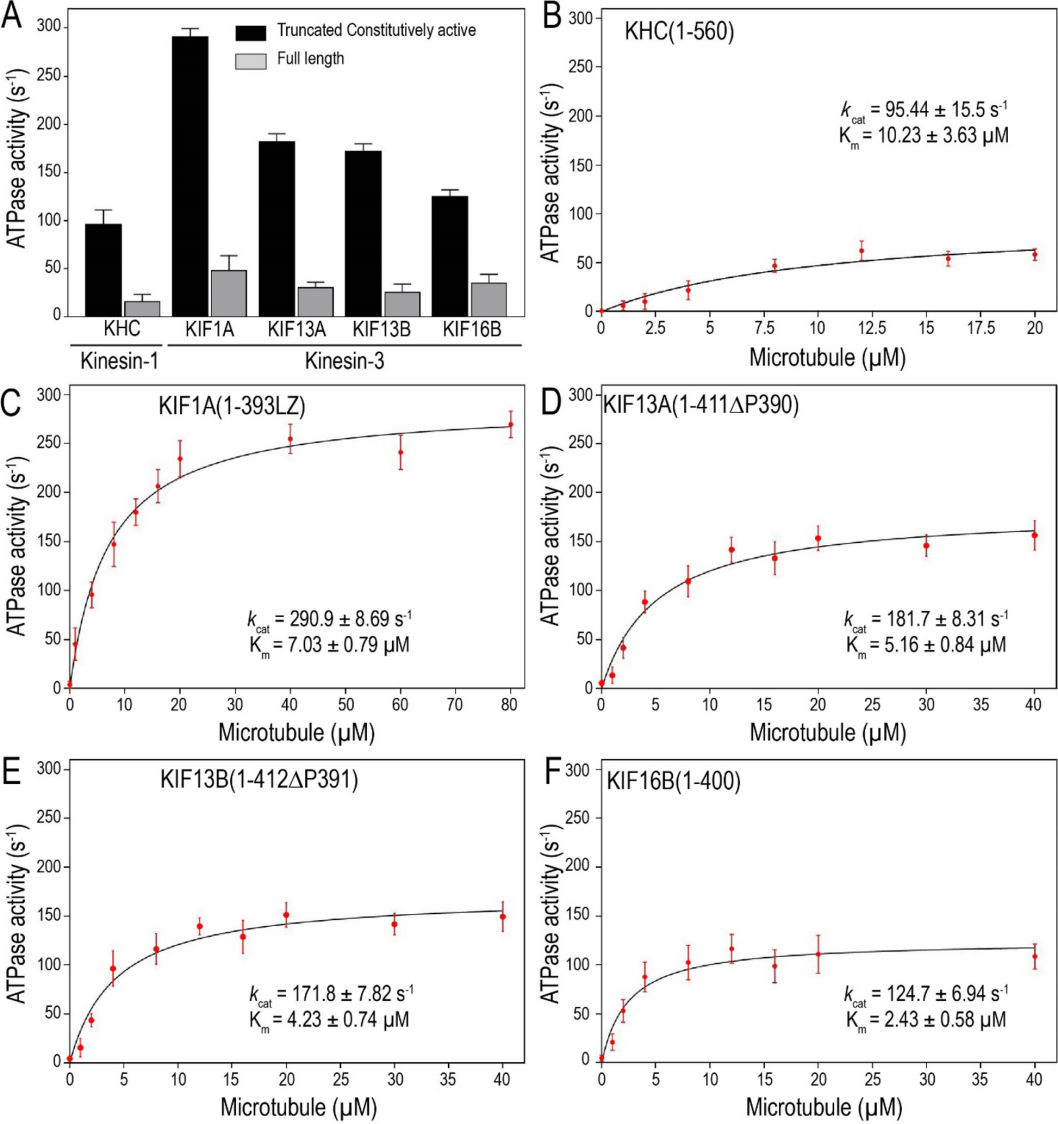
Kinesin-3 motors exhibit high ATPase activity and differential microtubule affinity. MT-stimulated ATPase activity of full-length and truncated constitutively active motors was measured using Sf9-purified proteins. **A** Comparison of ATPase activity between full-length and constitutively active kinesin motors. ATPase activity for full-length motors was measured at MT concentration that had highest ATPase activity for the respective constitutively active motor. **B–F** Plots showing ATPase activity against varied concentrations of MTs for **B** constitutively active kinesin-1 motor, KHC (1–560) and kinesin-3 motors **C** KIF1A(1-393LZ), **D** KIF13A(1-411ΔP), **E** KIF13B(1-412ΔP), and **F** KIF16B(1–400) and fit to Michaelis–Menten equation. Maximal turnover (*k*_cat_) and MT affinity (*K*_m_) parameters were determined using GraphPad Prism. Error bars represent mean ± SD. Data presented from three independent experiments

The assay with full-length KHC showed low basal ATPase activity even after adding MTs (Fig. 2A; Additional file 2: Table S1) [42, 43]. Consistent with the fact that kinesin-1 predominantly exists in a compact autoinhibited conformation, the tail domain folds back to interact with the motor domain directly [44–46]. This folded tail-to-head intramolecular interaction precludes the motor-MT association and subsequent ATP hydrolysis and holds the motor in an inactive folded conformation [47–50]. In contrast, constitutively active dimer, KHC(1–560), displayed approximately tenfold higher activity following the addition of MTs (Fig. 2B; Table 1). The determined *k*_cat_ and *K*_m_ were 95.44 ± 15.5 s^−1^ and 10.23 ± 3.63 µM, respectively, which agrees with previous reports [4, 44, 51, 52]. The ratio of the speed of kinesin-1 to its rate of MT-stimulated ATP hydrolysis yields a step size of ~ 8.54 nm and stoichiometry of 1.07 step/ATP hydrolyzed, suggesting a tight coupling between their chemical and mechanical cycles [6, 7].

A similar analysis using bacterially purified KHC(1–560) (Additional file 1: Fig. S1) exhibited deficient activity, indicating that majority of the motors were inactive. This finding again lends support to the importance of purifying kinesin motors from the eukaryotic expression system rather than from the prokaryotic system. Together, these results support the general autoinhibition mechanism in the field proposed for kinesin-1 and that the stepping rate of kinesin-1 is directly proportional to the speed of ATP hydrolysis.

#### Full-length kinesin-3 motors exhibit low ATPase activities

Next, we measured the ATPase activity of kinesin-3 family motors with and without MTs. Analogous to kinesin-1, the addition of MTs to full-length kinesin-3 motors did not stimulate their ATP hydrolysis (Fig. 2A; Additional file 2: Table S1). This observation is consistent with non-cargo-bound full-length kinesin-3 motors that exist in an autoinhibited state. Autoinhibition of motors prevents unnecessary hydrolysis of cellular ATP and interference with cargo trafficking on the MT. For kinesin-3, studies have shown that intramolecular interaction between the NC and CC1 domains keeps the motor in a monomeric, autoinhibited state [18, 53–60]. However, the detailed mechanism of kinesin-3 autoinhibition is still poorly understood.

#### KIF1A dimers exhibit the highest ATP turnover rates

Unlike full-length motors, the addition of MTs to constitutively active, dimeric kinesin-3 motors remarkably stimulated their ATPase activity, which is 1.3- to threefold higher than kinesin-1 (Fig. 2C–F). The rate of ATP hydrolysis for dimeric active KIF1A(1-393LZ) motor [17, 18] was found to be *k*_cat_ 290 ± 9 s^−1^, which is 3 times faster than the activity of KHC(1–560) (Fig. 2C; Table 1). The determined high *k*_cat_ is in close agreement with the estimated stepping rate (~ 301.29 s^−1^) with a step size of ~ 8.28 nm and stoichiometry of 1.0 step/ATP, suggesting a tight coupling between chemical and mechanical cycles. A similar step size of ~ 8 nm has been reported recently for KIF1A and UNC-104 motors [23]. Together, these results explain the fast velocity reported for this motor [17, 18, 28].

The measured ATPase activity is considerably higher than pioneering work from Hirokawa lab that demonstrated an ATP turnover rate of 110 ± 5 s^−1^ for a monomeric KIF1A [25, 26]. Similarly, an elegant and detailed experimental analysis of active UNC104 dimers showed a lower ATP turnover rate of 100 s^−1^/head [28]. Recent work from Zaniewski et al. also reported a significantly lower *k*_cat_ 115 s^−1^ for a stable KIF1A(1–406) dimers, suggesting a significant fraction of the molecules may be inactive, presumably due to motors purified from bacterial expression system [52]. The determined higher MT affinity of KIF1A(1-393LZ) (*K*_m_ 7.03 µM) compared to KHC(1–560) (*K*_m_ = 10.23 µM) agrees with previous studies on KIF1A/*Ce*Unc104 [17, 26, 28].

#### KIF13 dimers show higher ATPase activity

Contrary to full-length KIF13 motors, the addition of MTs to the dimeric active KIF13A(1-411ΔP) and KIF13B(1-412ΔP) motors strongly stimulated their ATPase rates with *k*_cat_ of 181.7 s^−1^ and 171.8 s^−1^, respectively (Fig. 2D,E; Table 1). These rates match their measured in vitro velocities (1.55 µm/s and 1.36 µm/s) and estimated stepping rates of 194.19 s^−1^ and 170.16 s^−1^, respectively [17, 18, 49]. Similar to KIF1A, the ratio of KIF13A and KIF13B motor velocities with their rates of ATP hydrolysis generates a step size of 8.53 and 7.91 nm, respectively, yielding a stoichiometry of ~ 1, suggesting a tight relation between ATP hydrolysis and motor stepping. Additionally, the measured ATPase rate constants of KIF13 motors were twice the rate of kinesin-1, corresponding to the observed difference in their velocities [18, 30, 49]. However, earlier studies using the truncated KIF13B(1–368) motor containing a catalytic motor domain and neck linker exhibited MT-stimulated ATP turnover rate 45 s^−1^ and *K*_m_ of 0.1 µM [61]. The observed low catalytic activity was presumably due to motor truncation lacking the dimerization domain (NC) and purified from bacterial expression system.

Similarly, previous MT-stimulated ATPase measurements for KIF13B full-length and motor truncation after FHA domain showed low ATP turnover rates (*k*_cat_ = 0.3 s^−1^ and *k*_cat_ = 4.6 s^−1^, respectively) [58], suggesting motors were probably in the autoinhibited state. Similarly, recent ATPase measurements of KIF13B truncations (including ΔP391) purified from bacterial expression system, showed lower activities compared to kinesin-1 [56]. Together, these results suggest that KIF13A and KIF13B motors have higher (~ 1.9- and 1.8-fold higher, respectively) ATPase activity than kinesin-1 motor and complement their measured velocities in vitro.

#### KIF16B dimers show high ATPase activity

The addition of MTs to KIF16B(1–400) triggered the ATP turnover rate *k*_cat_ (124.7 ± 6.94 s^−1^), which is approximately 1.3 fold higher than kinesin-1 but lower than KIF13 (1.4-fold) and KIF1A (2.3-fold) motors (Fig. 2F; Table 1). The ratio between the measured motor velocity and rate of ATP turnover resulted in an average step size of 7.61 nm/ATP, which implies a tight coupling between ATP hydrolysis and mechanical stepping. Additionally, KIF16B motor showed strongest MT affinity (*K*_m_ = 2.43 µM) compared to KIF13A (*K*_m_ = 5.16 µM), KIF13B (*K*_m_ = 4.23), KIF1A (*K*_m_ = 7.03 µM), and KHC (*K*_m_ = 10.23 µM) motors. These binding affinities correlate with their measured difference in landing rates [17, 18].

The binding affinity of kinesin-3 motors is influenced mainly by the positively charged lysine residues in the K-loop, a family-specific insert in the loop12 of the kinesin-3 motor domain (Additional file 1: Figs. S5A and S6). Crystal structure studies of KIF1A motor bound to microtubules revealed that the motor utilizes microtubule-binding loops, loop11, and loop12, in an alternative manner to switch its interaction with microtubule during ATPase cycle. In which conformational changes due to ATP hydrolysis withdraw loop11 from the microtubule and then engage loop12 with the microtubule [62]. Further detailed analysis using K-loop mutants with a varying number of lysine residues showed that the positively charged lysine residues in the K-loop dramatically increased their affinity to MTs [25, 26]. Though KIF16B motor contains only three lysine residues in the K-loop compared to six in KIF1A, the motor showed significantly higher MT affinity is interesting and requires future investigation. Together, our data suggest that despite kinesin-3 motors sharing a highly conserved motor domain, it's fine-tuning at the molecular level yields diverse motility outputs.

Our results provide the first comprehensive chemomechanical basis for high velocity and superprocessive motility of kinesin-3 family motors [17, 18]. We also show for the first time that the rate of ATP hydrolysis for kinesin-3 motors remarkably agrees with their stepping rate, suggesting a tight coupling between chemical and mechanical cycles. A characteristic feature of a processive motor is its ATPase cycle, which is gated by its ADP release and very slow in the absence of MTs. This ADP release is greatly accelerated by MTs, which consequently accelerates ATP turnover ~ 50 times as reported for kinesin-1 [42, 43, 63]. Based on these chemomechanical properties and structural similarities, we propose that kinesin-3 motors follow a similar transition state sequence, as demonstrated for kinesin-1. The truncated motors used in the ATPase assays consist of only the core catalytic motor domain and NC domain, critical for motor dimerization. Regions beyond the NC domain are known to regulate motor activity by inhibiting ATPase activity or preventing MT-binding or motor dimerization [16, 18, 33, 49, 58, 61]. Therefore, kinesin-3 motors’ ability to hydrolyze ATP at higher rates is intrinsic to their motor domains.

One possibility that can impact kinesin-3 motility is having a different sequence of chemomechanical states altogether than kinesin-1. However, this seems unlikely to explain kinesin-3 superprocessivity because kinesin motors share a highly conserved motor domain with > 65% sequence similarity. An alternative possibility is differences in specific rate constants in the ATP turnover cycle to influence motor speed and superprocessivity. Indeed, a recent chemomechanical analysis of KIF1A motor suggested that the rates of rear-head detachment, leading head attachment, and a strong MT interaction in the weakly bound state contribute to high velocity and superprocessivity of kinesin-3 motors [52]. The fact that all kinesin-3 motors have an average run length of 10 µm (Fig. 1; Table 1) with varying velocities suggests that faster motors will take a short time to travel 10 µm and so will have a higher *k*_off_ and thus higher *K*_m_. Based on these findings, we propose that differential MT-binding affinities with consequent robust ATPase activities are evolutionarily designed to render kinesin-3 family motors with unique mechanical outputs. To the best of our knowledge, this is the first comprehensive analysis of kinesin-3 motors that provides direct biochemical demonstration paralleling their observed high velocities, longer run lengths, and strong MT affinity. These findings open up many interesting questions of biological significance across the field.

### Kinesin-3 motor velocities inversely correlate to their microtubule-binding affinity

**Fig. 3:**
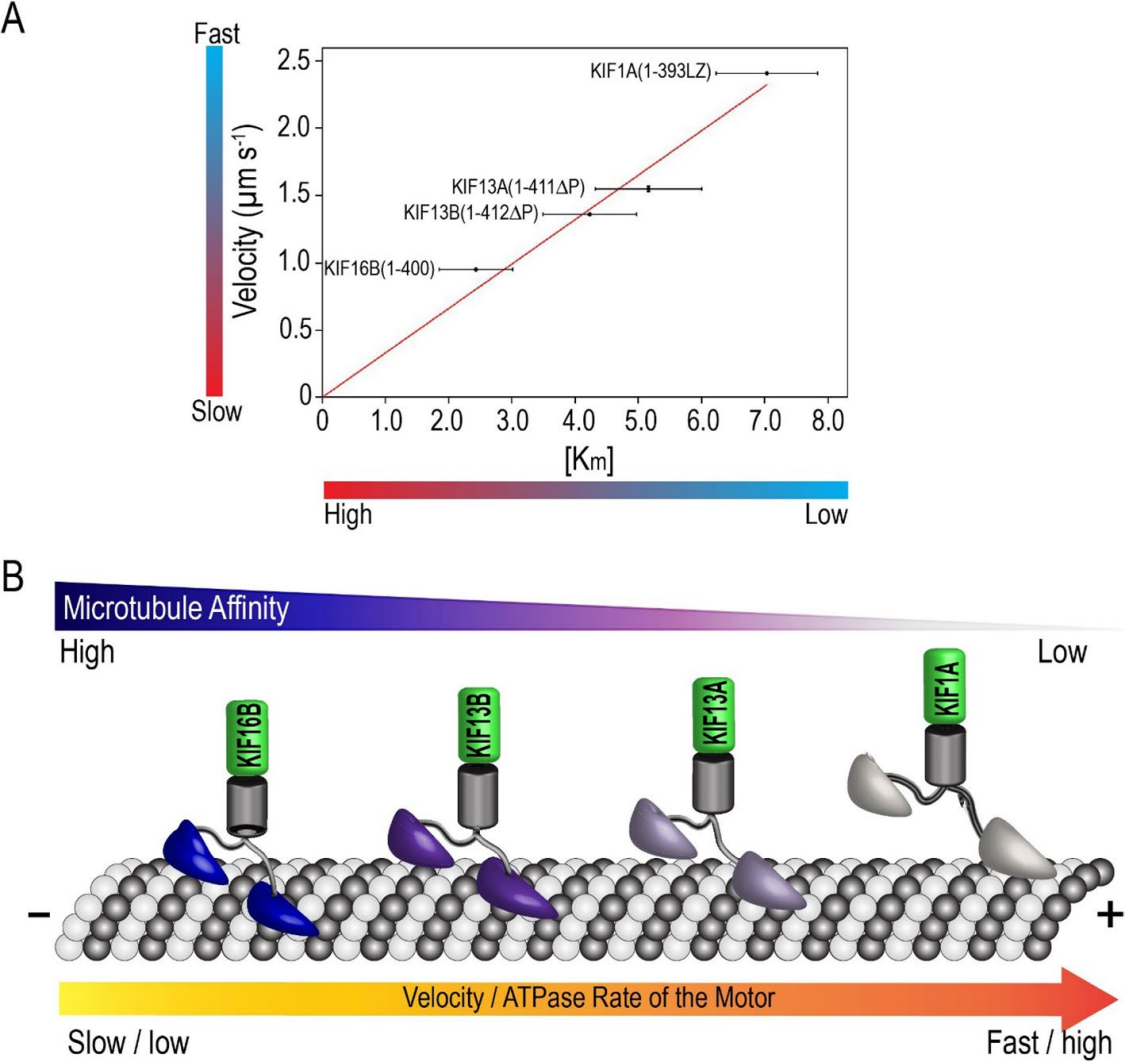
Kinesin-3 motor velocity inversely correlates with microtubule affinity. **A** The measured velocity of kinesin-1 and kinesin-3 motors in single-molecule motility assays was found to be inversely proportional to the MT-binding affinity obtained from ATPase analysis. **B** Cartoon diagram showing kinesin-3 family (KIF1A, KIF13A, KIF13B, and KIF16B) motors are robust ATPases, complementing with their measured velocities. These velocities inversely correlate with their differential microtubule-binding affinity

The velocities of kinesin-3 motors are tightly coupled with the rate of ATP hydrolysis, and interestingly, these velocities (KIF1A > KIF13A > KIF13B > KIF16B) showed an inverse correlation with their MT-binding affinities (KIF16B > KIF13B > KIF13A > KIF1A) (Fig. 3). As shown in Fig. 1, all kinesin-3 motors have a similar mean run length of ~ 10 µm, but their measured velocities inversely correlate with their MT-binding affinities (Table 1). For instance, KIF1A motors with faster velocity (2.41 µm s^−1^) and lower MT-binding affinity (*K*_m_ = 7.03 µM) take a shorter time of 4.38 s to run a distance of 10 µm, so they have a high MT detachment rate (*k*_off_ = 0.23 s^−1^). By contrast, KIF16B motors with slow velocity (0.95 µm s^−1^) and high MT-binding affinity (*K*_m_ = 2.43 µM) take a longer time of 9.83 s to cover the same 10 µm distance, hence have a low *k*_off_ (0.10 s^−1^). Similarly, KIF13 family motors, KIF13A and KIF13B with intermediate velocities (1.55 ± 0.02 µm s^−1^ and 1.36 ± 0.01 µm s^−1^, respectively) and moderate MT-binding affinities (*K*_m_ = 5.16 µM and *K*_m_ = 4.23 µM, respectively), take modest time scale of 6.61 ± 2.6 s and 8.06 ± 2.4 s, respectively, to cover a distance of 10 µm, so the intermediate *k*_off_ (0.15 s^−1^ and 0.12 s^−1^, respectively). Interestingly, our recent in silico analysis using the coupled Brownian motor model also predicted a similar trend (KIF1A > KIF13 > KIF16B) that is observed experimentally [64].

One hypothesis is that electrostatic interactions between the positively charged K-loop and the negatively charged MT surface contribute to the motor’s affinity for the MT, landing rate, and diffusion [17, 25, 26, 65, 66]. However, the K-loop of KIF1A contains six lysine residues, and the motor showed the lowest affinity for the MT than KIF13A, KIF13B, and KIF16B motors that have only three lysine residues (Additional file 1: Fig. S5; Table 1). Alternatively, the loop8, a third microtubule-binding region that does not change its conformation or position relative to the microtubule during ATPase turnover, interacts with the microtubule independent of its nucleotide state (Additional file 1: Fig. S6) [65, 67]. Recent molecular-dynamics simulations and binding energetic calculations reveal that the loop8 region establishes multiple engagements with the negatively charged H12 segment of β-tubulin and possibly provides extended MT affinity for kinesin-3 motors [19, 68]. Specifically, the measured higher MT-binding affinity for KIF13A and KIF13B motors are possibly due to arginine residue at R167 and R168, respectively, rather than a conserved lysine residue (K163 in KIF1A) (Additional file 1: Fig. S7A). Similarly, for KIF16B, the determined high MT-binding affinity, presumably due to additional arginine (R165 and R166) and lysine (K181) residues in loop8 (Additional file 1: Fig. S7A). Arginine and lysine are largely exposed to the protein surface and play critical roles in protein stability and affinity through electrostatic interactions. Most importantly, arginine residue enables multiple stable electrostatic interactions compared to lysine owing to its guanidinium group. Additionally, arginine residue generates more stable interactions due to its higher aqueous pKa (13.8) than lysine (10.53) residue [69, 70].

To test this, we mutated one of the arginine residues (R167C) in loop8 of KIF1A, which is critical for establishing stable interaction with negatively charged glutamate residue (E420) at the C-terminal tail region of tubulin subunits (Additional file 1: Fig. S6) while generating processive motility [15, 62, 67]. R167C is a heterozygous missense mutation found in patients suffering from Spastic Paraparesis [15]. Relative to the wild-type motor, COS-7 cells expressing mutant motors showed peripheral accumulation, albeit with less efficiency (Additional file 1: Fig. S7B). To further quantify its ability to take processive steps along the MT, we performed a CAD cell processivity assay [18]. Whereas differentiated CAD cells expressing wild-type motors showed substantial accumulation to the neurite tip, cells expressing mutant (R167C) motors showed significantly reduced tip accumulation (Additional file 1: Fig. S7C, D). To substantiate, we performed in vitro motility assays to measure their motility properties at the single molecule level. Wild-type motors showed uniform superprocessive motility along the MT surface (Additional file 1: Fig. S7E, F). However, mutant motors exhibited one-dimensional back-and-forth motion along the MTs (Additional file 1: Fig. S7E, F) and a significant decrease in the number of motors landing on the MTs, compared to wild-type motors (Additional file 1: Fig. S7G).

To determine the direct effect of mutation R167C on KIF1A microtubule affinity, we measured the enzyme kinetics using purified motors. Consistent with the motility data (Additional file 1: Fig. S7G), the mutant exhibited a significant decrease in the rate of ATP hydrolysis (~ 33%) and microtubule affinity (~ 1.5 times), compared to wild-type motors (Additional file 1: Fig. S7H, I) [71]. The residue R167 establishes multiple stable interactions with MT in ATP and ADP states [72]. Therefore, mutation R167C significantly affects motor-MT interaction, thereby affecting KIF1A motion along the MT. Additionally, the equivalent mutations (R167C) in other members of the kinesin-3 family, KIF13B (K172A) and KIF16B (R173A), resulted in a similar decrease in the rate of ATP hydrolysis and microtubule affinity (Additional file 1: Fig. S7H, I). Together, these results suggest that akin to R167 (KIF1A), its equivalent residues in other kinesin-3 motors make hydrogen bonding with negatively charged E420 at the tubulin tail region and contribute to the microtubule affinity and processive motility.

We further assessed the contribution of positively charged residues in the loop8 to the differential microtubule-binding by mutating other positively charged residues (K161A, K163A, and R169A) in KIF1A and measuring their ATPase kinetics using purified motors. Similar to R167C, the ATPase analysis of K161A and K163A mutants also showed a significant decrease in the ATP hydrolysis rates and affinity for the microtubules (Additional file 1: Fig. S7H, I), consistent with the previous work that conversion of all the positively charged residues in loop8 to alanine (K161A/R167A/R169A/K183A) strongly affected MT-binding affinity [15, 62, 71]. We did not measure the ATPase activity of the R169A mutant because the motor showed very low expression levels even after 2 days in mammalian cells and failed to express in Sf9 cells despite several attempts. Collectively, these results suggest that individual positively charged residues in the loop8 contribute substantially to the differential microtubule-binding affinity for the kinesin-3 motors, which is critical for establishing an inverse correlation between the rate of ATP hydrolysis and microtubule affinity.

Together, we hypothesize that although kinesin family motors share a highly conserved motor domain, a fine balance between the rate of ATP hydrolysis and its affinity for the MT is critical for generating family-specific motility outputs. Indeed, previous studies on kinesin and myosin motors have suggested that the rate of ATP hydrolysis and nature of interaction with the MT directly regulates family-specific mechanical outputs [4, 40]. The best example that illustrates such a correlation would be the studies done on sprinters of the land, cheetah [73]. Analogies between kinesin-3 unique mechanical outputs and a sprinting cheetah: powerful muscle strength (high ATPase activity), firm traction (K-loop and loop8), and decreased ground contact time (lower MT affinity). However, detailed kinetics of ATP turnover cycle is needed to understand quantitative differences in the transition rates of ATPase cycle. Collectively, we demonstrate that a fine balance between the rate of ATP hydrolysis and microtubule-binding affinity enables kinesin-3 motors with novel mechanical outputs.

### Kinesin-3 motors influence microtubule bending in vitro

**Fig. 4:**
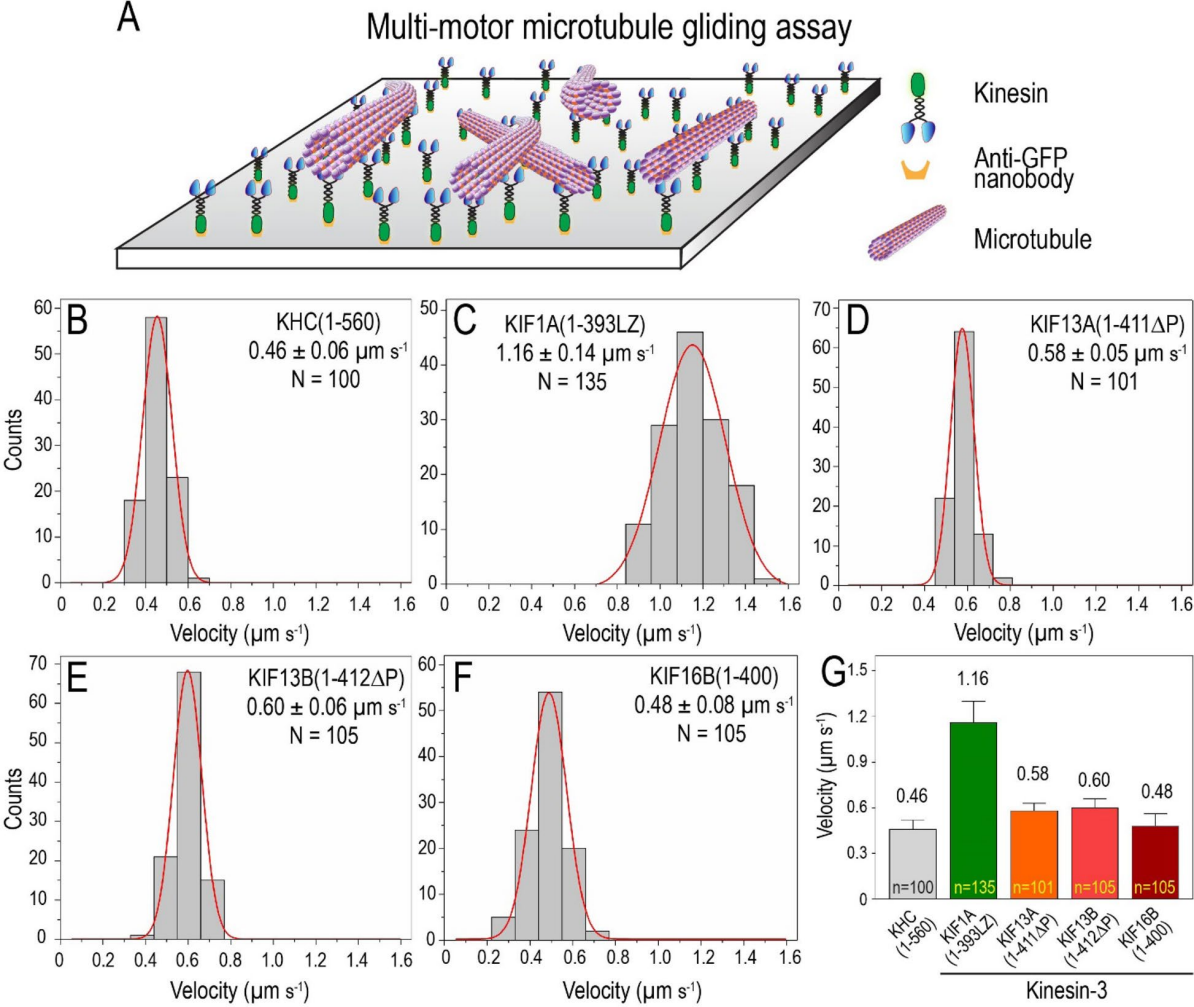
Kinesin-3 motors glide microtubules faster. Collective motor properties of kinesin-3 motors in MT gliding assays. **A** Schematic representation of MT gliding assay. C-terminal mCitrine-tagged truncated constitutively active kinesin-3 motors were attached to a glass surface coated with GFP nanobodies. Subsequently, fluorescently labeled MTs were introduced into the flow chamber. **B–F** Histograms showing velocity of MT sliding by kinesin-1 motor **B** KHC (1–560) and kinesin-3 motors **C** KIF1A(1-393LZ), **D** KIF13A(1-411ΔP), **E** KIF13B(1-412ΔP), and **F** KIF16B(1–400) were plotted and fit to Gaussian distribution. Average gliding velocities are indicated as mean ± SEM on top-right or left corner. N, number of motility events analyzed across three independent experiments. **G** Gliding velocities for kinesin-1 and kinesin-3 motors were plotted as mean ± SEM

In vitro single-molecule motility properties correspond to measured biochemical properties of kinesin motors. Next, we wanted to investigate the collective behavior of kinesin-3 motors, which can be studied in vitro by MT gliding assay [34, 74, 75]. In the gliding assay, constitutively active kinesin-1 or kinesin-3 motors, purified from Sf9 cells, were immobilized on the glass surface inside the motility flow chamber (Fig. 4A). As these purified motors contain a C-terminal mCit (mCitrine)-tag, we used GFP nanobodies [76] to attach motors to the glass surface. Rhodamine-labeled MTs were polymerized and sheared with a syringe before infusing into the motility flow chamber. The gliding motion of microtubules was recorded under TIRF illumination. The landing of MTs on the glass surface coated with motor proteins showed smooth MT sliding. We also observed efficient MT crossover without any noticeable hindrance in their gliding velocities [77, 78]. We manually tracked multiple gliding MTs for each kinesin motor and plotted them as histograms to determine their average velocities (Fig. 4B–G).

**Fig. 5:**
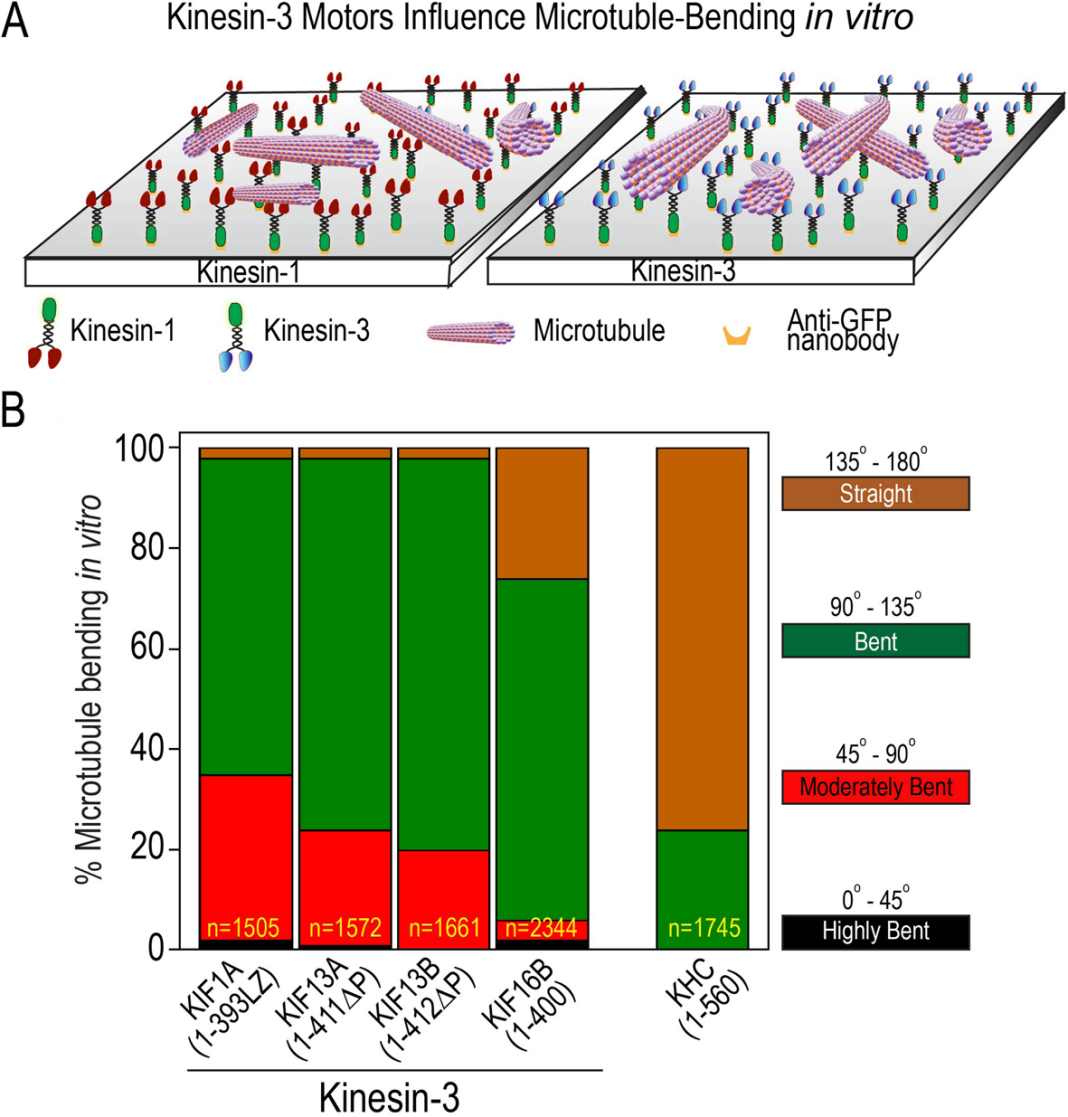
Kinesin-3 motors induce frequent microtubule bending in multi-motor gliding assay. **A** Cartoon representation of constitutively active kinesin-3 motors influence significant microtubule bending in multi-motor gliding assay in vitro compared to kinesin-1 motor. **B** Bar graph indicating the percent MT bending in multi-motor gliding assay with constitutively active kinesin-3 and kinesin-1 motors. MTs ≤ 20 μm were considered for bending analysis. *n* values represent number of bending events analyzed for angle measurement. Values from three independent experiments

For kinesin-1, KHC(1–560) motor displayed smooth translocation of MTs over long distances with an average speed of 0.46 ± 0.06 µm s^−1^ (Fig. 4B; Additional file 1: Fig. S8A; Table 1; Additional file 3: Movie S1), as shown previously [28, 79–81]. Akin to kinesin-1, members of kinesin-3 motors purified from Sf9 cells showed smooth MT sliding, albeit with higher velocities. KIF1A(1-393LZ) showed robust MTs sliding with an average speed of 1.16 ± 0.14 μm s^−1^ (Fig. 4C, Additional file 1: Fig. S8B; Table 1; Additional file 3: Movie S2), which is ~ 2.5-fold higher than the average velocity determined for kinesin-1. The fast velocity observed for KIF1A is comparable to previously reported gliding velocity of 0.91 ± 0.09 µm s^−1^ measured using protein expressed in mammalian cells [82]. However, similar studies using motors purified from bacterial expression system showed velocities ranging from 0.05 to 0.35 µm s^−1^ [12, 83, 84], presumably due to lack of specific chaperone system or premature termination of protein synthesis leading to misfolded or truncated proteins [31]. Surprisingly, we noticed that MTs propelled by KIF1A(1-393LZ) motors induced frequent MT bending. Quantitative analysis revealed that ~ 90% of microtubules showed bending in the range of 45 to 135°, significantly higher than kinesin-1, which ranges from 90 to 180° (Fig. 5A,B).

MT gliding analysis of constitutively active KIF13 family members, KIF13A(1-411ΔP) and KIF13B(1-412ΔP), displayed vigorous MT sliding with intermittent bending. The average velocity of sliding MTs was determined to be 0.58 ± 0.05 µm s^−1^ and 0.60 ± 0.06 µm s^−1^ for KIF13A(1-411ΔP) and KIF13B(1-412ΔP), respectively (Fig. 4D,E; Additional file 1: Fig. S8 C-D; Table 1; Additional file 3: Movie S3, S4), as shown previously [59, 85, 86]. Similar to KIF1A, quantitative analysis showed ~ 90% of microtubules bending in the range of 45 to 135° for both KIF13A(1-411ΔP) and KIF13B(1-412ΔP) motors (Fig. 5A,B). Lastly, analysis of KIF16B(1–400) also showed uniform MT gliding, with occasional MT bending. The tracking analysis yielded an average velocity of 0.48 ± 0.08 µm s^−1^ (Fig. 4F; Additional file 1: Fig. S8E; Table 1; Additional file 3: Movie S5) [87], which is much slower than other kinesin-3 members but still comparable to the velocity determined for kinesin-1. Unlike other kinesin-3 motors, KIF16B showed ~ 70% microtubule bending between 90–135° **(**Fig. 5A,B).

Together, the data suggest that our low-density MT (~ 0.5 MTs/μm^2^) gliding assays using purified constitutively active kinesin-3 family motors influence MT bending without affecting their gliding velocities. Interestingly, the observed MT bending does not require pinning of the leading end or any other physical barrier as reported previously [88–90]. Therefore, understanding the mechanism that influences MT bending requires further characterization.

### Kinesin-3 motors influence microtubule bending in vivo

**Fig. 6:**
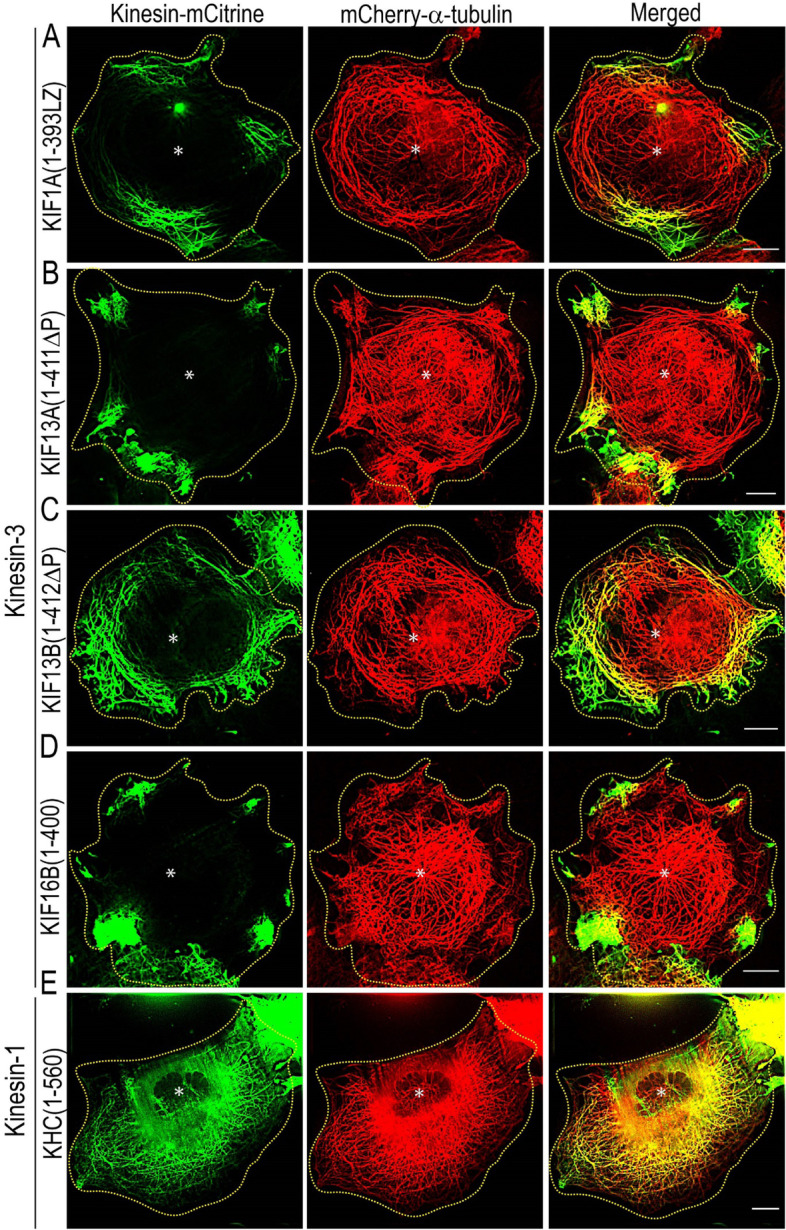
Kinesin-3 motors induce microtubule bending in cells*.* COS-7 cells were cotransfected with plasmids coding for mCherry-α-tubulin and individual members of constitutively active wild-type kinesin-3 **A** KIF1A(1-393LZ), **B** KIF13A(1-411ΔP), **C** KIF13B(1-412ΔP), and **D** KIF16B(1–400), and kinesn-1 **E** KHC(1–560) motors. Yellow dotted line indicates the cell boundary, and asterisk indicates the nucleus. Scale bars, 10 μm

To investigate the underlying mechanism of microtubule bending at the cellular level, we performed live-cell imaging in cells expressing kinesin-3 or kinesin-1 motors. We imaged microtubule architecture in COS-7 cells expressing mCherry-α-tubulin under TIRF illumination. Cells coexpressing constitutively active wild-type kinesin-3 or kinesin-1 motors with mCherry-tubulin displayed varying MT decoration and architecture (Fig. 6). For kinesin-1, KHC(1–560) motor, a significant population (~ 95%) of cells showed a characteristic radial array of microtubules with shallow MT decoration. Only a small (5%) population of cells showed microtubule bending (Additional file 1: Fig. S9A). In contrast, the majority (50–60%) of the cell population expressing constitutively active kinesin-3 motors showed strong MT decoration and MT bending (Additional file 1: Fig. S9A). MT bending analysis of cells expressing kinesin-3 motors revealed ~ 95% of bending between 45 and 135°, which is significantly higher than kinesin-1, ranging from 90 to 180° (Additional file 1: Fig. S9B). The extent of microtubule decoration agrees with the previously observed significant difference in MT landing and binding affinities in vitro [17]. Together these results suggest that kinesin-3 motors influence microtubule bending in vivo.

### The K-loop influences microtubule bending in vivo

The K-loop, a conserved, hallmark insert in loop 12 of kinesin-3 motor domain, contains several positively charged lysine residues (Additional file 1: Fig. S5A) that play essential roles in motor-MT interaction in the ADP state [25, 26, 62]. The detailed biochemical and biophysical analysis has demonstrated that the K-loop influences motors affinity for the MT, landing rate, and diffusion through an electrostatic interaction with negatively charged E-hook at the C-terminal tubulin dimers [17, 25, 26, 65, 66]. However, the influence of K-loop in MT bending in kinesin-3 motors has never been explored. Thus, we examined its role in MT bending using constitutively active dimeric kinesin-3 wild-type KIF1A(1-393LZ), KIF13A(1-411ΔP), KIF13B(1-412ΔP), and KIF16B(1–400) and its K-loop mutants [17]. As a control, we used KHC(1–560), a well-characterized constitutively active dimeric kinesin-1 motor [17, 18].

**Fig. 7:**
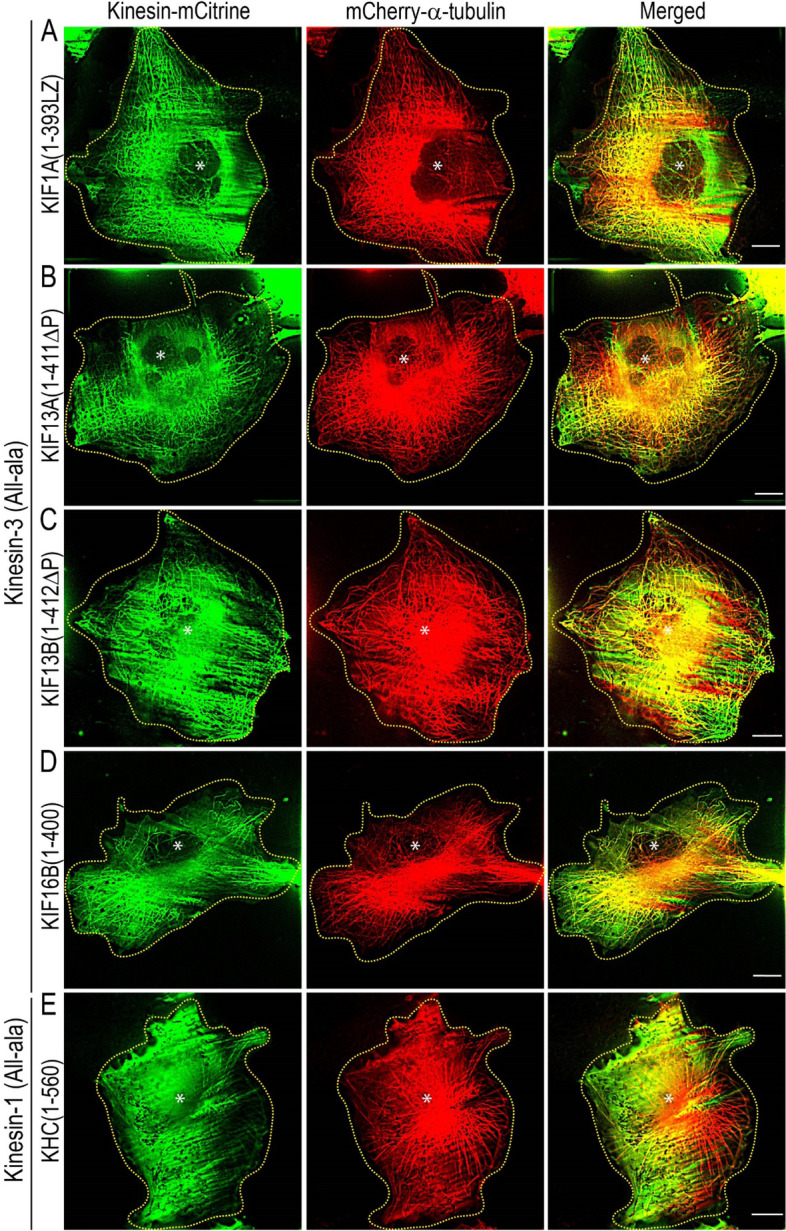
Positively charged lysine resides in the K-loop influence microtubule bending in cells. COS-7 cells were cotransfected with plasmids coding for mCherry-α-tubulin and individual members of constitutively active alanine (All-ala) mutants of kinesin-3 **A** KIF1A(1-393LZ), **B** KIF13A(1-411ΔP), **C** KIF13B(1-412ΔP), and **D** KIF16B(1–400), and kinesn-1 **E** KHC(1–560), in which positively charged lysine residues in the K-loop were mutated to alanine as described previously [17]. Yellow dotted line indicates the cell boundary, and asterisk indicates the nucleus. Scale bars, 10 μm

To dissect the contribution of the K-loop in MT bending at a molecular level, we expressed K-loop mutants (All-ala), in which all lysine residues in the K-loop of each motor mutated to alanine residues (Additional file 1: Fig. S5B) [17]. Consistent with previous findings that mutation of lysine-to-alanine residues in the K-loop, all the kinesin-3 motors significantly lost their ability to bind MTs and remained largely cytoplasmic. In addition, these cells showed no significant MT bending; instead showed radial MT arrays extending towards the cell periphery, with occasional bending similar to cells expressing kinesin-1 (Fig. 7A–D; Additional file 1: Fig. S9B). Similarly, the conversion of a single lysine residue to alanine in the K-loop of kinesin-1 showed no change in its ability to interact with MTs (Fig. 7E; Additional file 1: Fig. S9B). Together, these results suggest that positively charged lysine residues in the K-loop influence kinesin-3 driven MT bending in cells.

**Fig. 8:**
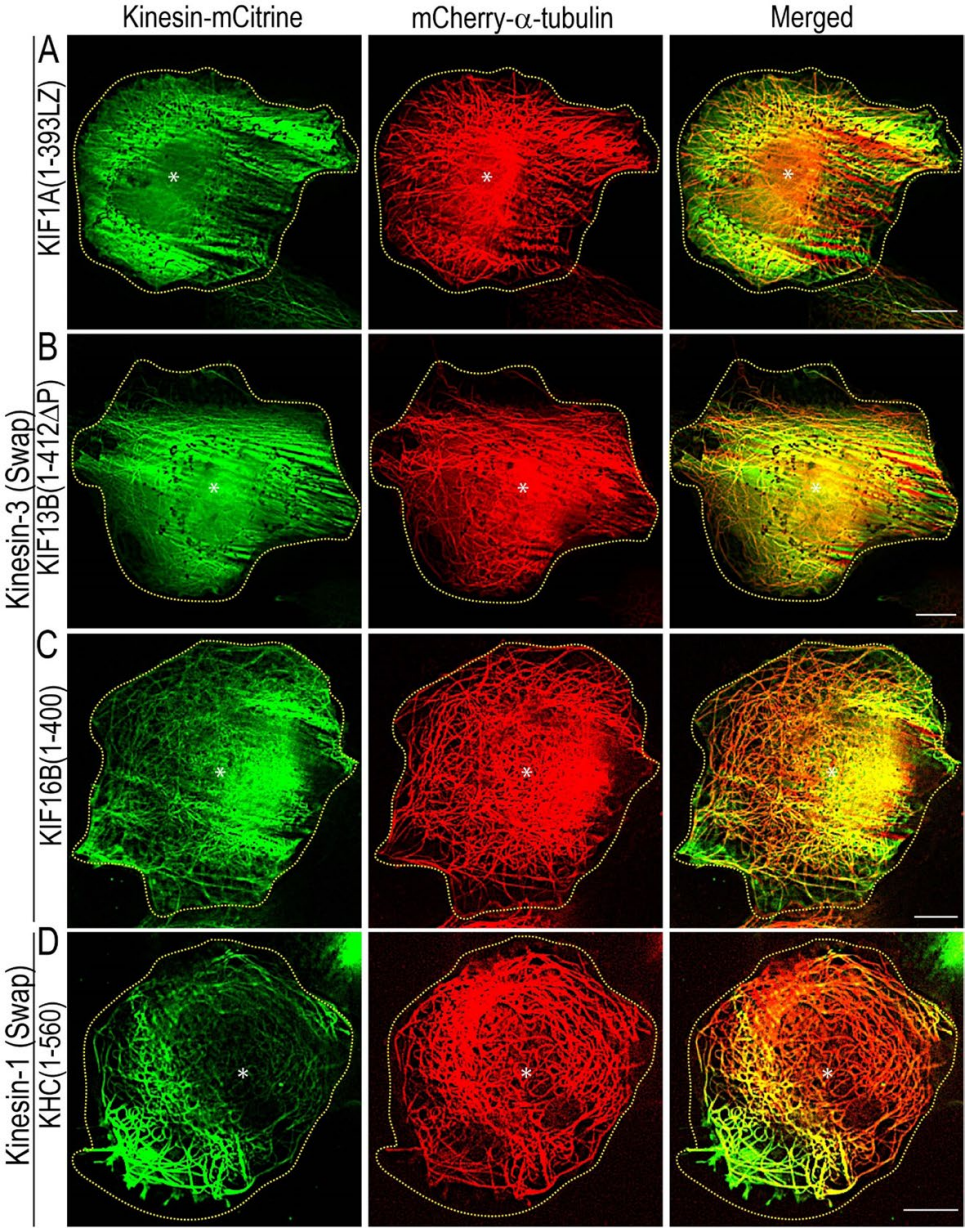
Positively charged lysine resides in the K-loop influence microtubule bending in cells. COS-7 cells were cotransfected with plasmids coding for mCherry-α-tubulin and individual members of constitutively active swap mutants in which the K-loop of each kinesin-3 motors **A** KIF1A(1-393LZ), **B** KIF13B(1-412ΔP), and **C** KIF16B(1–400) were replaced with that of kinesin-1 and **D** the K-loop of kinesin-1 was replaced with that of KIF1A. Yellow dotted line indicates the cell boundary, and asterisk indicates the nucleus. Scale bars, 10 μm

To further explore the specificity of K-loop in MT bending, we expressed swap mutants, in which the K-loop sequence of each kinesin-3 motor is replaced with that of kinesin-1 (Additional file 1: Fig. S5C) [17]. KIF13A and KIF13B are homologs and share ∼62% amino acid identity [91]. Their constitutively active dimeric versions showed identical motility properties and regulation mechanisms; we only included the analysis for the KIF13B motor for the sake of simplicity. The cells expressing kinesin-3 swap mutants not only significantly lost their ability to decorate MTs but also considerably lost MT bending ability (Fig. 8A–C). The extent of MT bending was similar to kinesin-3 All-ala and wild-type kinesin-1 motors (Additional file 1: Fig. S9B). Paradoxically, the generation of kinesin-1 swap mutant by substituting the corresponding loop 12 of kinesin-1 with that of KIF1A showed a marked increase in MT decoration and bending (Fig. 8D; Additional file 1: Fig. S9B), which is comparable to wild-type kinesin-3 motors.

### The K-loop influences microtubule bending in vitro

**Fig. 9:**
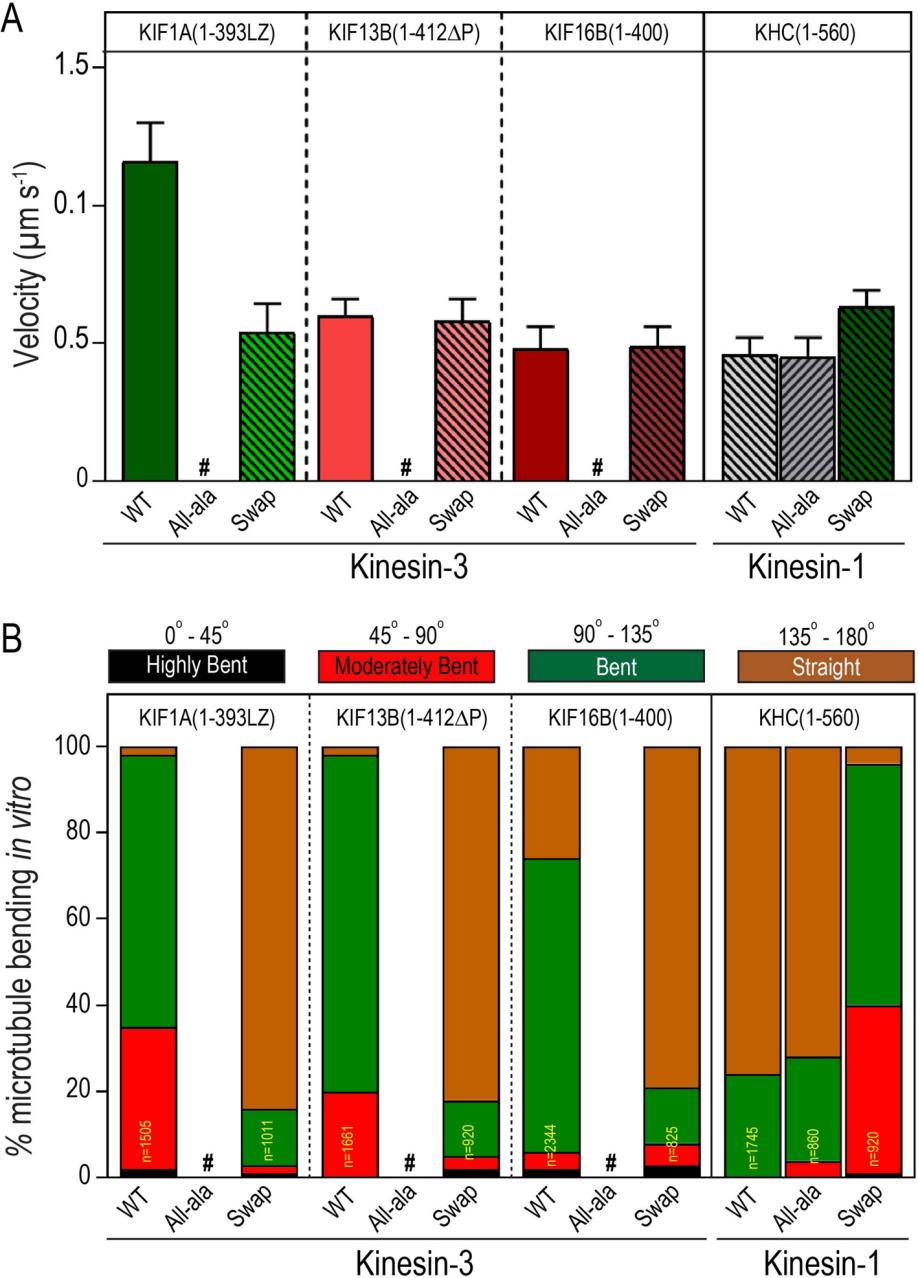
K-loop, a family-specific insert in the loop12 of Kinesin-3 motor domain, influences microtubule bending. **A** Microtubule gliding velocities for wild-type and K-loop mutants (All-ala and Swap) of kinesin-3 (KIF1A, KIF13B, and KIF16B) and kinesin-1 (KHC) motors were plotted as mean ± SD. **B** Microtubule-bending analysis of wild-type and K-loop mutants (All-ala and Swap) of kinesin-3 (KIF1A, KIF13B, and KIF16B) and kinesin-1 (KHC) motors in multi-motor microtubule gliding assay. #, no motility events were observed

As kinesin-3 all-alanine and swap mutants failed to exhibit microtubule bending and kinesin-1 swap mutant showed significant microtubule bending in vivo, we decided to test these results in a purified system using in vitro microtubule gliding assays. Assays with kinesin-3 All-ala mutants exhibited very few loosely bound microtubules on the surface, resulting in no microtubule gliding events (Fig. 9A,B). MT gliding assay with KIF1A All-ala mutant is provided as a representative video (Additional file 3: Movie S6). These results agree with our previous single-molecule motility assays of kinesin-3 All-ala mutants that failed to show processive motion along the microtubule surface [17]. However, gliding analysis with kinesin-3 Swap mutant exhibited smooth and uniform microtubule motion at an average speed between 0.49 and 0.58 µm s^−1^ with occasional microtubule bending (~ 10%) similar to wild-type kinesin-1 (Fig. 9A,B). MT gliding assay with KIF1A Swap mutant is provided as a representative video (Additional file 3: Movie S7). In contrast, experiments with kinesin-1 Swap mutant displayed uniform and smooth microtubule gliding at an average velocity of 0.63 ± 0.01 µm s^−1^ with significant microtubule bending (~ 90%), which is comparable to KIF1A wild-type motor (90%) (Fig. 9; Additional file 3: Movie S8). Together, these results demonstrate that the K-loop not only increases affinity for the MT but also influences MT bending.

MTs are long cylindrical rigid structures that can withstand compressive loads to resist and balance the tensional forces in cells and their network supports a myriad of biological processes [92]. MTs exhibit divergent behavior in various cellular processes ranging from mechanical support to intracellular transport to cellular signaling. Particularly, MT bending has been shown to alter growing plus-end dynamics, localization, and its role in targeting and biochemical signaling [93–95]. MT bending on both short and long length scales is ubiquitous in mammalian and fungal cells due to large forces generated by the action of molecular motors [92, 96–100]. MT bending has also been observed in cell-free and obstacle-free systems through an unknown mechanism [101]. Despite MT architecture playing prime biological roles, the mechanism of MT bending and its in vivo significance remains poorly studied. More recently, detyrosination of α-tubulin has been shown to regulate the MT bending and stiffness in beating cardiomyocytes. Interestingly, increased detyrosination prompted MT bending, stiffened the myocyte, and implicated cardiac and muscular dystrophy [102]. However, understanding the physiological significance of this interesting phenomenon requires detailed biophysical and structural characterization, which may further provide important insights on MT bending observed in nerve cells undergoing degeneration [103–105].

## Conclusions

In summary, we successfully purified recombinant full-length and constitutively active kinesin-3 family motors using Sf9-baculovirus expression system. These purified proteins are of high quality, as demonstrated by their robust biochemical ATPase, multi-motor MT gliding, and single-molecule motility. The measured high ATPase activity and strong microtubule affinity of kinesin-3 family motors correlate to their high velocity and superprocessivity. Additionally, positively charged amino acid residues clustered in the loop8 of kinesin-3 motor domain largely contribute to the determined differential microtubule-binding affinities. These microtubule-binding affinities inversely correlate to the velocities of kinesin-3 motors. Furthermore, kinesin-3 multi-motor MT gliding analysis has displayed uniform MT sliding and smooth crossing over with significant MT bending than kinesin-1 motor. This microtubule bending is largely influenced by K-loop, a family-specific insert in the loop-12 of kinesin-3 motor domain. To our knowledge, this is the first comprehensive demonstration of kinesin-3 family motors with diverse mechanical outputs. Thus, we propose that kinesin-3 motors are fine-tuned at the molecular level to endow diverse mechanical outputs critical for myriad cellular processes.

## Materials and methods

### DH10Bac competent cell preparation

LB broth supplemented with kanamycin and tetracycline was inoculated with overnight grown DH10Bac primary culture and incubated at 37 °C until OD reached 0.4–0.6. The cells were harvested by spinning at 8000 rpm for 10 min at 4 °C and resuspended in 100 ml competency buffer (60 mM CaCl_2_, 10 mM PIPES, 15% glycerol, pH 7.0). After 30 min of incubation on ice, cells were collected by centrifuging at 4000 rpm for 10 min, 4 °C. This final pellet was resuspended in competency buffer and snap-frozen into small aliquots in liquid nitrogen before storing at − 80 °C.

### Generation of recombinant bacmid

Recombinant expression of a specific protein in Sf9 cells requires generation of a recombinant bacmid. A key step in generating recombinant bacmid is the transposition of gene-of-interest into the bacmid [106]. Though generation of recombinant bacmid using commercially available bac-to-bac kits (ThermoScientific) is simple and works well for small inserts, recombination is difficult for large-size inserts. Members of the kinesin-3 superfamily are large proteins with a molecular weight ranging from 140 to 200 kDa. Moreover, it would be expensive, especially when working with multiple samples. Thus, we generated recombinant bacmids containing full-length and constitutively active kinesin-1 and kinesin-3 motors by employing bac-to-bac methodology in-house. The coding sequences for kinesin-1 [full-length, KHC-mCit-Flag and constitutively active, KHC(1–560)-mCit-Flag] and kinesin-3 [full-length, KIF1A-mCit-Flag, KIF13A-mCit-Flag, KIF13B-mCit-Flag and KIF16B-mCit-Flag and constitutively active, KIF1A(1-393LZ)-mCit-Flag, KIF13A(1-411ΔP390)-mCit-Flag, KIF13B(1-412ΔP391)-mCit-Flag and KIF16B (1–400)-mCit-Flag] were sub-cloned into pFastBac backbone plasmid using PCR amplification.

The above plasmids were transformed into DH10Bac competent *E. coli* carrying bacmid genome and helper plasmid and incubated at 37 °C for site-specific homologous recombination to generate recombinant bacmids with gene-of-interest. For full-length motors, 12–16 h of incubation time was required for successful homologous recombination between pFastBac [containing C-terminal mCitrine-Flag (mCit-Flag)-tagged full-length versions of kinesin-1 and kinesin-3 motors] and bacmid in DH10Bac cells due to their large insert size. For constitutively active versions of kinesin-1 and kinesin-3 motors, incubation for approximately 6–8 h was sufficient.

In general, blue-white screening for recombinant bacmid often gives false positive colonies, and contamination of empty bacmid will result in low-to-no expression. Also, selection of recombinant bacmids usually requires screening a large number of white colonies (> 10) by isolating bacmid DNA using expensive bacmid isolation kits. Therefore, we performed colony-PCR using bacmid-specific primer pair and screened more than 40 and 20 colonies each for full-length and constitutively active motors, respectively. Subsequently, we purified only the colony-PCR-positive recombinant bacmids using PureLink plasmid isolation kit (Invitrogen) and confirmed the transposition using bacmid-specific primers. Additionally, we performed PCR with a combination of bacmid-specific and gene-specific primers. These purified recombinant bacmids were used for transfection and recombinant protein production in Sf9 insect cells. Together, these results suggest that transposition of large inserts can be achieved in-house just by incubating for a longer time in DH10Bac competent cells. This can be applied for recombination of other large proteins in bacmid. Moreover, a simple and economical colony-PCR with bacmid-specific primers can be used for the initial blue/white screening of recombinant bacmids.

### Sf9 cell culture, transfection, and recombinant baculovirus production

Sf9 cells were a kind gift from Dr. Thomas Pucadyil (Indian Institute of Science Education and Research, Pune, India). Sf9 cells were maintained in Sf-900/SFM medium (Invitrogen) at 28 °C. For transfection, cells were seeded into 35-mm plates at a density of 4.5 × 10^5^. Next day, these cells were transfected with a mixture of 1 μg bacmid DNA and 6 μl Cellfectin transfection reagent (Invitrogen) as per the manufacturer’s instruction. Since all our constructs were tagged with fluorescent protein mCitrine, we could monitor the cells for expression after 48 h of transfection. The supernatant contains the first generation of recombinant baculovirus (P0 virus) were collected after 72 h of transfection. The P1 viruses were further amplified and used to infect large-scale suspension cultures. Three days post-infection, supernatant containing amplified recombinant baculoviruses were collected by centrifugation at 500 g for 10 min and stored at − 80 °C and used for all future infection and protein purification experiments.

### One-step purification of kinesin-3 motors

Transfection of individual recombinant bacmids carrying C-terminal mCit-Flag–tagged kinesin-1 or kinesin-3 motors resulted in robust expression of respective motor proteins as determined by Western blotting and epifluorescence microscopy. The expression of recombinant protein reached its maxima between 48 and 72 h. A large portion (~ 85%) of recombinant motor protein was found in soluble form, whereas only a small fraction (~ 15%) of the protein was found in pellet fraction.

For purification, 30 ml suspension culture of Sf9 cells expressing recombinant protein (~ 72 h post-infection) were harvested by spinning at 500 g, 4 °C for 10 min. Cell pellet was lysed in lysis buffer (20 mM HEPES, 200 mM NaCl, 4 mM MgCl_2_, 0.5% IGEPAL and 7% Sucrose, pH 7.5) supplemented with 5 mM DTT, 5 µg/ml aprotinin, 5 µg/ml leupeptin, and 5 µg/ml PMSF. Lysate was clarified at 150,000 g for 30 min at 4 °C, and this high-speed supernatant was then incubated with anti-FLAG M2 affinity resin for 2–3 h at 4 °C. The resin was washed thrice with wash buffer (20 mM HEPES, 300 mM KCl, and 2 mM MgCl_2_, pH 7.5) supplemented with 2 mM DTT, 5 µg/ml aprotinin, 5 µg/ml leupeptin, and 5 µg/ml PMSF. Protein was eluted with 100 µg/ml FLAG peptide by incubating overnight at 4 °C. The purified protein was found to be stable at 4 °C for more than a week, with no significant loss of activity. The protocol yielded a recombinant protein of high purity and sufficient quantity for further analysis. The typical yield of purified proteins was ranged from 15 to 20 mg per liter of Sf9 culture with no visible impurities. All the characterization assays were performed using freshly prepared protein samples unless otherwise stated. The homogeneity of the purified protein was further confirmed by size exclusion-high-performance liquid chromatography (SEC-HPLC) using Phenomenex SEC-3000 column with 1 × PBS as mobile phase.

### Circular dichroism (CD) spectroscopy

For CD measurements, the purified protein sample was analyzed at 200 nM concentration in 10 mM sodium phosphate, pH 7.0. The spectra was recorded from 190 to 250 nm at 0.1-nm interval and 50 nm min^–1^ using spectropolarimeter (Jasco-815). The average of three scans was used to determine the mean residue molar ellipticity [*θ*] (deg × cm^2^ × dmol^–1^) was calculated according to the formula:

$$\left[\theta \right]=\theta /\left({N}_{\mathrm{A}}\times d\times {C}_{\mathrm{M}}\times 10\right)$$

where *θ*, measured ellipticity in degrees; *N*_A_ is the number of amino acids per protein; *d* is the path length in centimeters; *C*_M_ is the molar protein concentration. The factor 10 originates from the conversion of the molar concentration to the dmol cm^–3^ concentration unit. The secondary structure estimation and folding status was performed on BeStSel program [107, 108].

### Bacterial purification of constitutively active kinesin-1

Constitutively active kinesin-1, KHC (1–560)-GFP-His plasmid was transformed into BL21 pLyS cells. The overnight grown primary culture was inoculated into 200 ml LB media along with antibiotics and incubated at 37 °C for 2 h. Once O.D reaches 0.4–0.6, culture was induced with 0.5 mM isopropyl β-d-1-thiogalactopyranoside (IPTG), and cells were grown at 22 °C overnight. After pelleting, the cells were lysed using B-PER™ reagent (Thermo Fisher Scientific) in the presence of protease inhibitors and processed further for purification using a standardized protocol.

### Cell culture, transfection, and fluorescence microscopy

COS-7 (monkey kidney fibroblast; American Type Culture Collection, Manassas, VA) cells were grown in DMEM supplemented with 10% (vol/vol) FBS (Thermo Fisher Scientific, USA) at 37 °C with 5.0% CO_2_. Cells were transfected/cotransfected with plasmid DNA of interest using Turbofect (Thermo Fisher Scientific, USA). Subsequent day, cells were lysed to prepare cell lysates or fixed for fluorescence microscopy. The mouse catecholaminergic cell line CAD [109] was grown in DMEM F12 with 10% (vol/vol) FBS (Thermo Fisher Scientific, USA) at 37 °C with 5.0% (vol/vol) CO_2_. Cells were prompted to differentiate by replacing with serum-free media and then transfected with plasmid DNA of interest using Lipofectamine 3000 (Thermo Fisher Scientific, USA). After 48 h, the cells were prepared for fluorescence microscopy.

For cell lysates, 24 h of post transfection COS-7 cells were washed, trypsinized, and lysed in ice-cold lysis buffer (LB; 25 mM 4-(2-hydroxyethyl)-1-piperazineethanesulfonic acid/KOH, 115 mM potassium acetate, 5 mM sodium acetate, 5 mM MgCl2, 0.5 mM ethylene glycol tetraacetic acid [EGTA], 1% [vol/vol] Triton X-100, pH 7.4) freshly supplemented with 1 mM phenylmethylsulfonyl fluoride and protease inhibitors (10 μg/ml leupeptin, 5 μg/ml chymostatin, 3 μg/ml elastatinal, 1 mg/ml pepstatin). The lysates were clarified by centrifugation at 16,000 × *g* at 4 °C and either it was immediately used for single-molecule assays or snap-freeze aliquots were frozen in liquid nitrogen and stored at − 80 °C until further use.

For fluorescence microscopy, 48 h of post transfection cells were washed once with warm phosphate buffer saline (PBS), fixed in 4% (vol/vol) paraformaldehyde (PFA) in PBS for 10 min and then mounted in Prolong Gold (Thermo Fisher Scientific, USA). Images were acquired on Nikon Eclipse Ti2-E, motorized automated inverted microscope, attached with Perfect Focus System, oil-immersion × 60 1.49 NA objective, Andor iXon Ultra 897 EMCCD camera, and NIS-Elements Advanced Research image acquisition software. To quantify motor accumulation, the average fluorescence intensity of the cell body and its neurite tip were measured using ImageJ software (National Institutes of Health, Bethesda, MD). The mean ± SEM for wild-type and mutant motors were plotted as the ratio of average fluorescence intensity in the neurite tip to that in the cell body using Prism 6 software (GraphPad Software, La Jolla, CA).

### Tubulin purification and labeling

Tubulin was purified from goat brain using the facility generously provided by Prof. Roop Mallik, TIFR, Mumbai, India, as described previously [110] and stored in liquid nitrogen until further use. Fluorescent labeling of tubulin was performed using protocol described previously with modifications [111]. Above purified tubulin was incubated with 20 molar excess NHS-rhodamine (Thermo Scientific) at 37 °C for 10 min. The reaction was terminated by adding an equal volume of potassium glutamate, and the mixture was loaded onto a Sephadex G50 column kept at 4 °C. The labeled tubulin was collected and cycled through a polymerization and depolymerization before snap freezing into small aliquots.

### Microtubule polymerization and single-molecule assays

MTs were polymerized from purified tubulin from goat brain in BRB80 buffer (80 mM 1,4-piperazinediethanesulfonic acid [PIPES]/KOH, pH 6.8, 1 mM MgCl_2_, 1 mM EGTA) supplemented with 1 mM GTP at 37 °C for 20 min. Polymerized MTs were stabilized by adding five volumes of prewarmed P12 buffer containing 20 μM Taxol and additional 5-min incubation at 37 °C. For labeled MTs, rhodamine-labeled tubulin and non-labeled tubulin was mixed at a 1:10 ratio and polymerized as described above.

### Single-molecule motility assay

All single-molecule motility assays were carried out at room temperature in a narrow flow cell (∼10 μl volume) prepared by attaching a clean #1.5 coverslip to a microscope slide with double-sided. Polymerized MTs (30–50 μm in length) were further diluted in P12 buffer (12 mM PIPES/KOH, pH 6.8, 1 mM MgCl_2_, and 1 mM EGTA supplemented with 10 μM Taxol) and then flowed into a flow cell and incubated for 5 min in humid chamber to adsorb onto the coverslip. Subsequently, coverslip surface was blocked by flowing 50 μl of blocking buffer (10 mg/ml bovine serum albumin in P12 buffer with 10 μM Taxol) and incubated for 20 min to prevent nonspecific binding of kinesin motors. Finally, a 50-μl motility mix consisting of purified motors, 25 µL of P12, 25 µL blocking buffer, 1 mM DTT, 2 mM ATP, 20 μg/μl glucose oxidase, 8 μg/μl catalase, and 1 M glucose, was added to the flow chamber and the ends were sealed with molten paraffin wax before imaging under TIRF illumination. Imaging was performed using total internal reflection fluorescence (TIRF) microscopy: Nikon Eclipse Ti2-E, motorized automated, equipped with Perfect Focus System, oil-immersion × 60 1.49 NA objective, Andor iXon Ultra 897 EMCCD camera, and NIS-Elements Advanced Research image acquisition software. Movies were acquired using 488 nm laser at 200 ms exposure with no delay.

For landing rate measurements, the amount of wild-type or mutant motors in the COS-7 lysates was first normalized by a dot-blot in which increasing volumes of COS-7 lysates were spotted onto nitrocellulose membrane. The membrane was air-dried and processed for immunoblotting for mCit-tag using anti-GFP antibody (A6455, Thermo Fisher Scientific, USA). The spots were quantified to normalize the motor concentration. Equal amounts of motors were added to flow cells and imaged at 20 fps. The number of motors landing on a MT was counted and then divided by the total length of the MTs and the recording time in order to obtain a landing rate with the units of events/micrometer/minute.

### ATPase assay

All ATPase assays were performed at room temperature as described previously for smooth muscle myosin [41]. Briefly, an assay mixture containing 10 nM purified kinesin motor, 1–80 μM polymerized MT, 1 mM DTT, and 20 mM ATP was set up in BRB80 and incubated for 2 h. At every 30-min time interval, a sample of 25 µl was collected and mixed with 25 µl of stop solution (500 mM EDTA, 10% SDS) to cease the reaction. After completion of all time points, 100 µl of developing solution (0.5% ammonium molybdate, 5 mg/ml ferrous sulfate) was added and absorbance was recorded at 655 nm within 15 min. For generating the standard curve, KH_2_PO_4_ solution was used. To determine the ATPase activity, the slope of phosphate release as a function of time was plotted. To convert nmol/min × µg to s^−1^, we divide with a constant which depends on the molecular weight of the motor protein that is used. To determine the *V*_max_ and *K*_m_, ATPase

$$\mathrm{Activity}\left(\mathrm{nmol}/\min\times\mathrm\mu\mathrm g\right)=\frac{\mathrm{Slope}\left(\mathrm{OD}/\min\right)\;\times\;\mathrm{Slope}\;\mathrm{of}\;\mathrm{standard}\;\mathrm{curve}\;\left(\mathrm{nmol}/\mathrm{OD}\right)}{\mu\mathrm{g}\;\mathrm{in}\;25\;\mu\mathrm{l}}$$

activity as a function of substrate concentration is used and fitted to a Michaelis–Menten equation. To determine ATP turnover rate (*K*_*cat*_) following equation is used.

$${K}_{cat}=\frac{{V}_{\mathrm{max}}}{\mathrm{Et}}$$

where Et, total enzyme concentration; *V*_max_, maximum enzyme activity. All the ATPase assays were performed in duplicates from three independent protein preparations.

### Gliding assay

Rhodamine-labeled MTs were used for gliding assay. Polymerized labeled MTs were sheared using a 26-G needle. Flow chamber was created using double-sided tape to attach coverslip to a glass slide. GFP nanobody (100 nM) was flown into the flow chamber and incubated for 30–45 min at room temperature. Then, 15 mg/ml casein in P12 buffer was passed to block nonspecific binding and incubated for 5 min. The purified motor was diluted in P12-casein with 2 mM ATP was flown and incubated for 20–30 min. Final MT mix containing sheared MTs, 2 mM ATP, 20 μg/μl glucose oxidase, 8 μg/μl catalase, and 1 M glucose in P12-casein was added to the flow chamber and imaged under TIRF illumination.

### Data analysis

For single-molecule motility and gliding assays, the data analysis was performed using a custom-written ImageJ plug-in (nih.gov). For single-molecule motility assays, fluorescently labeled individual motors were tracked manually frame-by-frame. Similarly, for gliding assays, translocation of fluorescently labeled MTs were tracked manually frame-by-frame. The number of events for each sample was plotted for velocity and run length as a histogram and fit to a Gaussian distribution. All the statistical analyses were performed using GraphPad Prism (8.0).

### Microtubule bending analysis

For detailed quantification of microtubule bending, we have set the following criteria and divided the microtubule bending into four categories based on their degree of bending: Straight (135–180°), Bent (90–135°), Moderately Bent (45–90°), and Highly Bent (0–45°). To measure microtubule bending angle in vitro and in vivo, we used built in angle tool in ImageJ software (nih.gov), which measures the angle between the first and second segment of a segmented line selection. Quantified results are presented as percent bar graphs.

## Supplementary Information

**Additional file 1: Figure S1.** Bacterial expression of KHC(1-560) resulted in inactive and degraded protein. **Figure S2.** Generation of recombinant bacmids for of kinesin-1 and kinesin-3 motors. **Figure S3.** Baculovirus-purified constitutively active kinesin-1 and kinesin-3 motors. **Figure S4.** *In vitro* microtubule-based single-molecule motility assays of constitutively active kinesin-3 motors. **Figure S5.** Amino acid sequence alignment of kinesin-3 and kinesin-1 K-loop and their mutants. **Figure S6.** Ribbon diagram of KIF1A motor domain interacting with tubulin subunits. **Figure S7.** Loop8 contributes to the strong microtubule-binding affinity for kinesin-3 motors. **Figure S8.** Multi-motor microtubule gliding analysis of kinesin-3 motors. **Figure S9.** Kinesin-3 motors influence microtubule bending *in vivo*.

**Additional file 2: Table S1.** Summary of ATPase properties of full-length kinesin-1 and kinesin-3 motors.

**Additional file 3: Movie S1.** Microtubule gliding assay of constitutively active kinesin-1 motor, KHC(1-560). **Movie S2.** Microtubule gliding assay of constitutively active kinesin-3 motor, KIF1A(1-393LZ). **Movie S3.** Microtubule gliding assay of constitutively active kinesin-3 motor, KIF13A(1-411ΔP). **Movie S4.** Microtubule gliding assay of constitutively active kinesin-3 motor, KIF13B(1-412ΔP). **Movie S5.** Microtubule gliding assay of constitutively active kinesin-3 motor, KIF16B(1-400). **Movie S6.** Microtubule gliding assay of KIF1A(1-393LZ) All-alanine mutant. **Movie S7.** Microtubule gliding assay of KIF1A(1-393LZ) Swap mutant. **Movie S8.** Microtubule gliding assay of KHC(1-560) Swap mutant.

**Additional file 4.** Uncropped blot for Fig. S1B.

**Additional file 5.** Uncropped blot for Fig. S3B.

**Additional file 6.** Uncropped blot for Fig. S3C.

## Data Availability

All data are available in the main text or the additional materials.
