# Supplementary material for: Kinesin-3 motors are fine-tuned at the molecular level to endow distinct mechanical outputs
Source: BMC Biol. 2022 Aug 10;20:177. doi: 10.1186/s12915-022-01370-8 (PMC9364601; doi:10.1186/s12915-022-01370-8)
Supplement: Supplementary file 1 — Additional file 1: Figure S1. Bacterial expression of KHC(1-560) resulted in inactive and degraded protein. Figure S2. Generation of recombinant bacmids for of kinesin-1 and kinesin-3 motors. Figure S3. Baculovirus-purified constitutively active kinesin-1 and kinesin-3 motors. Figure S4. In vitro microtubule-based single-molecule motility assays of constitutively active kinesin-3 motors. Figure S5. Amino acid sequence alignment of kinesin-3 and kinesin-1 K-loop and their mutants. Figure S6. Ribbon diagram of KIF1A motor domain interacting with tubulin subunits. Figure S7. Loop8 contributes to the strong microtubule-binding affinity for kinesin-3 motors. Figure S8. Multi-motor microtubule gliding analysis of kinesin-3 motors. Figure S9. Kinesin-3 motors influence microtubule bending in vivo. [file 12915_2022_1370_MOESM1_ESM.docx]

Additional Materials for

Kinesin-3 Motors are Fine-Tuned at the Molecular Level to Endow Distinct Mechanical Outputs

Pushpanjali Soppina^1, 2^, Nishaben Patel^1,3^, Dipeshwari J. Shewale^1^, Ashim Rai^3^, Sivaraj Sivaramakrishnan^3^, Pradeep K. Naik^2^ and Virupakshi Soppina^1^*

*Corresponding author. Email: [**vsoppina@gmail.com**](mailto:vsoppina@gmail.com)**;** [**vsoppina@iitgn.ac.in**](mailto:vsoppina@iitgn.ac.in)

**This PDF file includes:**

Additional file 1: Figure S1 to S9

**Legends for Movies S1 to S8**

**
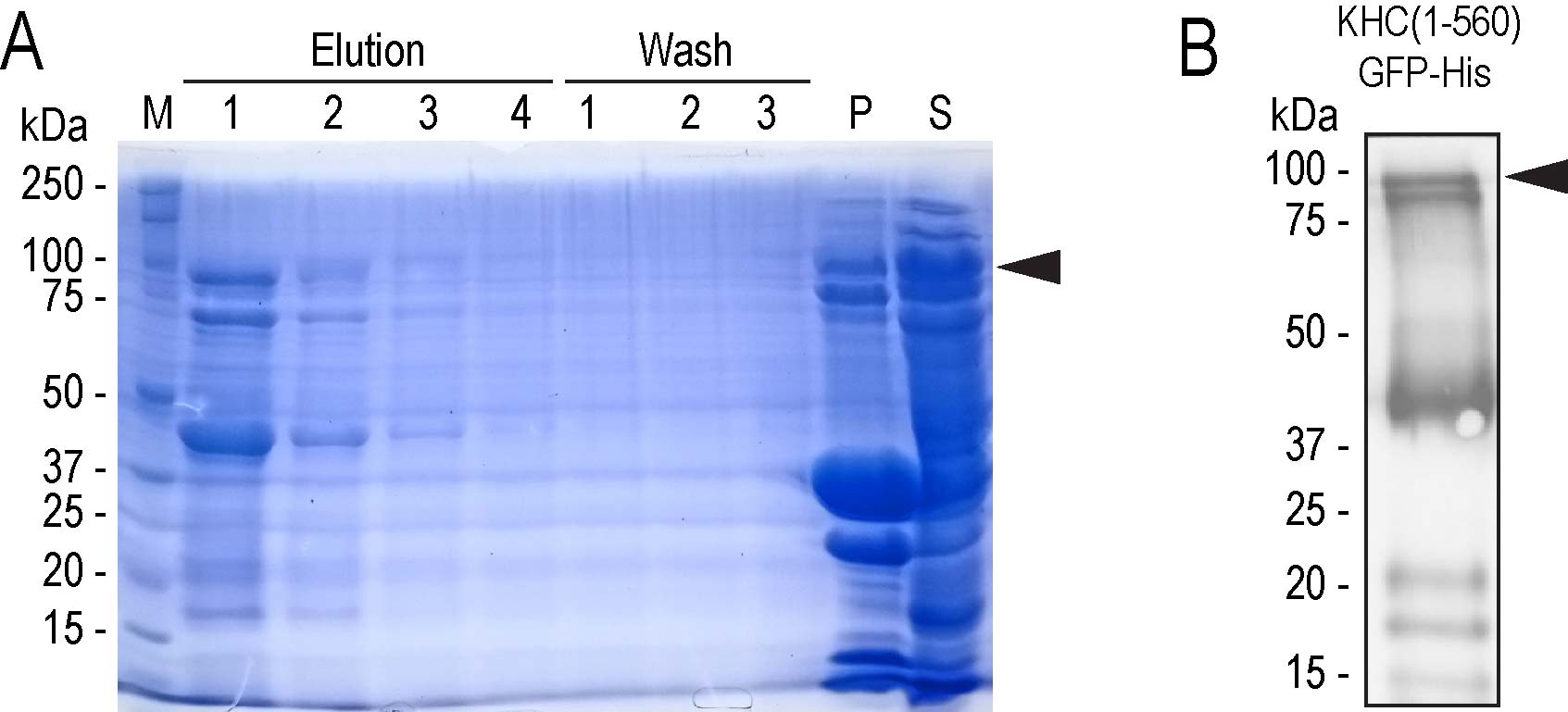
**

**Fig. S1. Bacterial expression of KHC(1-560) resulted in inactive and degraded protein. (A-B)** Coomassie gel and Western blot images showing purified KHC (1-560) GFP-His protein from bacterial expression system. (A) Coomassie gel represents purified protein fractions, protein standard (M), elution fractions (lane 2-5), washed fractions (lane 6-8), pellet (P), and supernatant (S) after bacterial lysis and high-speed centrifugation. Though the expected band size is ~80kDa, multiple bands can be seen in purified sample also. (B) For confirmation, elution fraction 1 was blotted with anti-His primary antibody. Again, multiple bands can be seen in Western blot, could be due to premature translation termination or degradation.

**
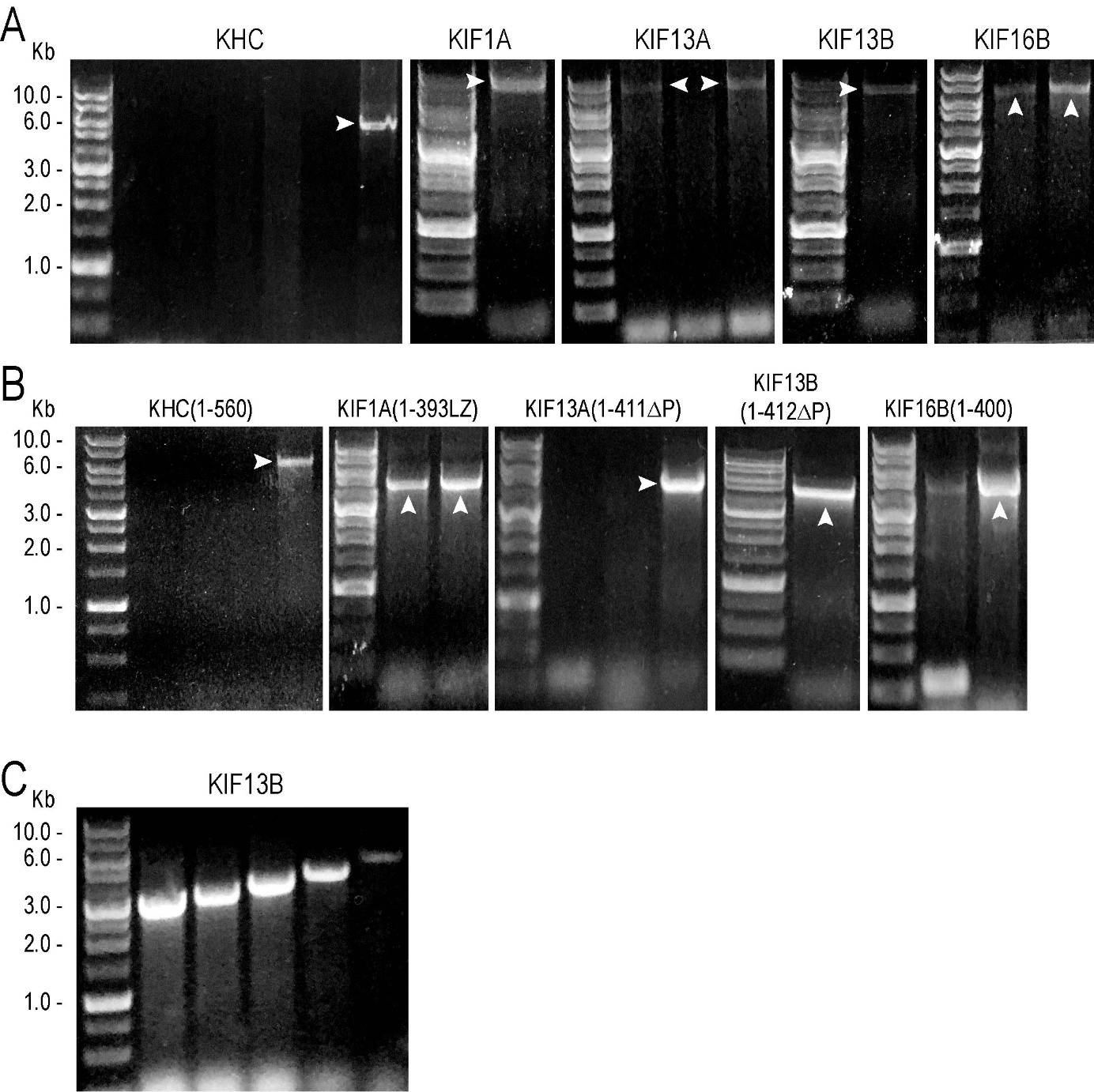
**

**Fig. S2. Generation of recombinant bacmids for kinesin-1 and kinesin-3 motors.** Recombinant bacmids were generated for full-length and truncated constitutively active kinesin-1 and kinesin-3 motors using bac-to-bac system. (A-B) Screening for positive recombinant bacmid for full-length (A) and constitutively active (B) motors via colony-PCR. Expected band for recombinant bacmid for each kinesin motor is indicated with white arrow head. (C) Representative gel for screening of bacmid with gene-specific (KIF13B) primers binding at different positions throughout the gene.

**
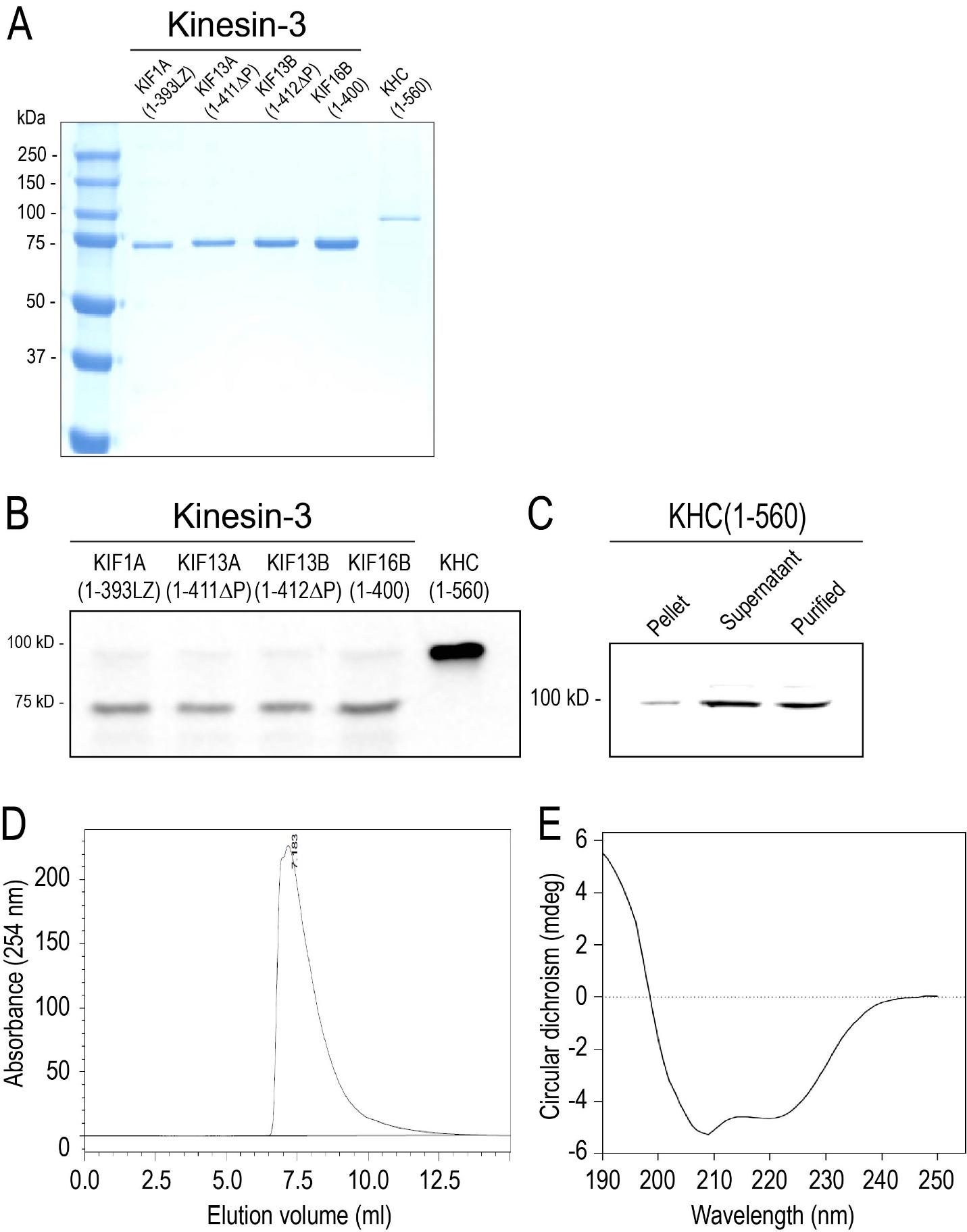
**

**Fig. S3. Baculovirus-purified constitutively active kinesin-1 and kinesin-3 motors.** (A) Coomassie-stained SDS-PAGE showing Sf9-baculovirus-purified truncated constitutively active kinesin-1 and kinesin-3 motors, as indicated on top. (B) Recombinant bacmids for constitutively active kinesin motors tagged with mCitrine-Flag were expressed in Sf9 cells. After 72 hrs of transfection, Sf9 cells were lysed and blotted for Flag using anti-Flag antibody to check their expression. (C) Pellet, supernatant and final purified product for KHC(1-560)-mCit-Flag were blotted with anti-Flag antibody, showing that motors expressed in Sf9 cells are largely cytoplasmic and soluble. (D) HPLC analysis of purified KIF1A(1-393LZ)-mCit-FLAG protein shows elution as a single peak. (E) Circular dichroism (CD) spectroscopy shows the helical propensities of the purified KIF1A(1-393LZ)-mCit-FLAG. The estimated secondary structures showed 48% of α-helix, 32% of β-structure and 19% others.

**
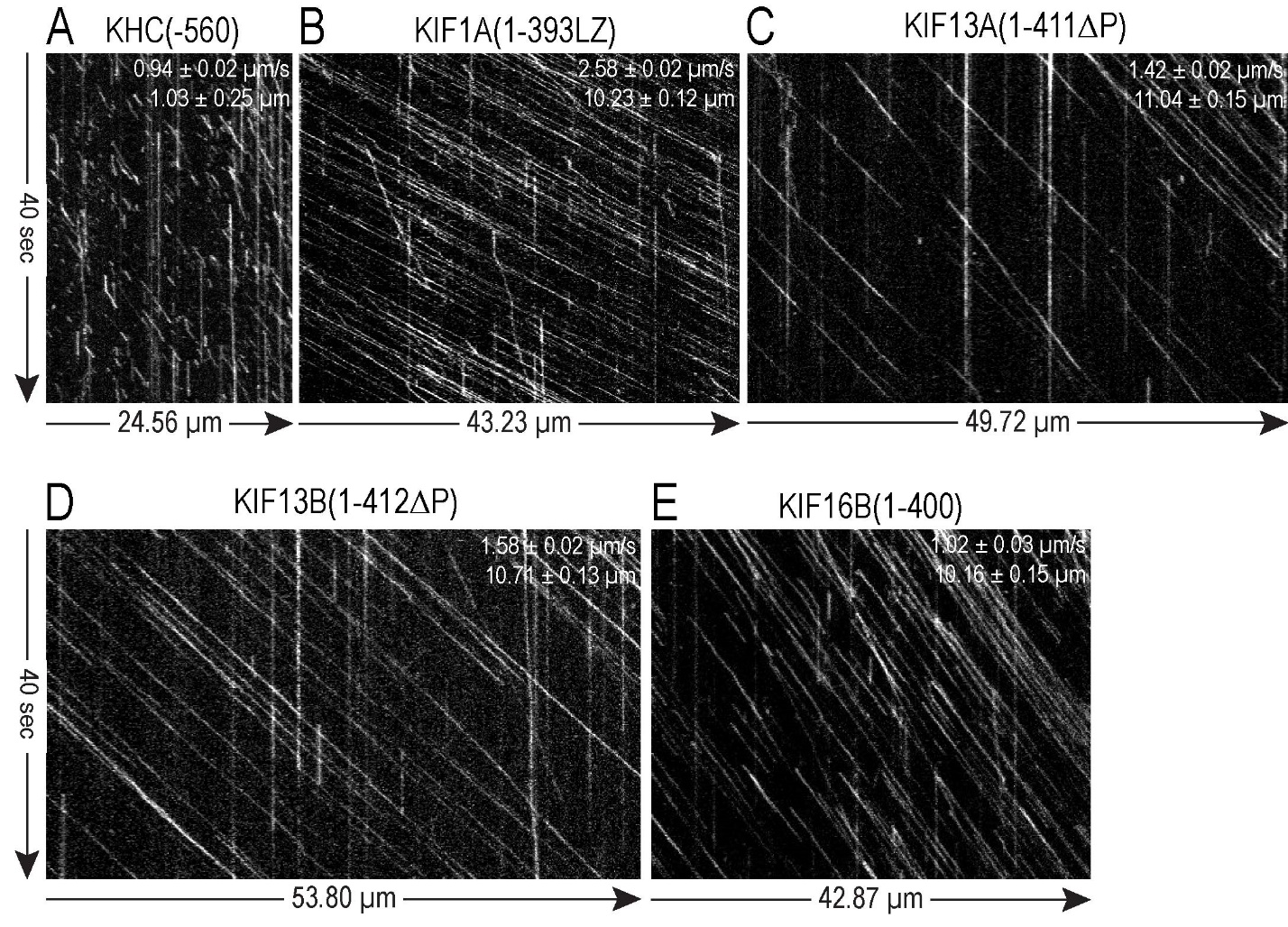
**

**Fig. S4. *In vitro* microtubule-based single-molecule motility assays of constitutively active kinesin-3 motors.** Single-molecule motility properties were analysed using fluorescently-tagged constitutively active kinesin motors purified from Sf9-baculovirus expression system. (A-E) Representative kymographs of individual kinesin motors walking processively (white lines) along the MT surface for (A) KHC(1-560), (B) KIF1A(1-393LZ), (C) KIF13A(1-411ΔP) (D) KIF13B(1-412ΔP) and (E) KIF16B(1-400),. Time on y-axis (vertical arrow) and distance on x-axis (horizontal arrow).

**
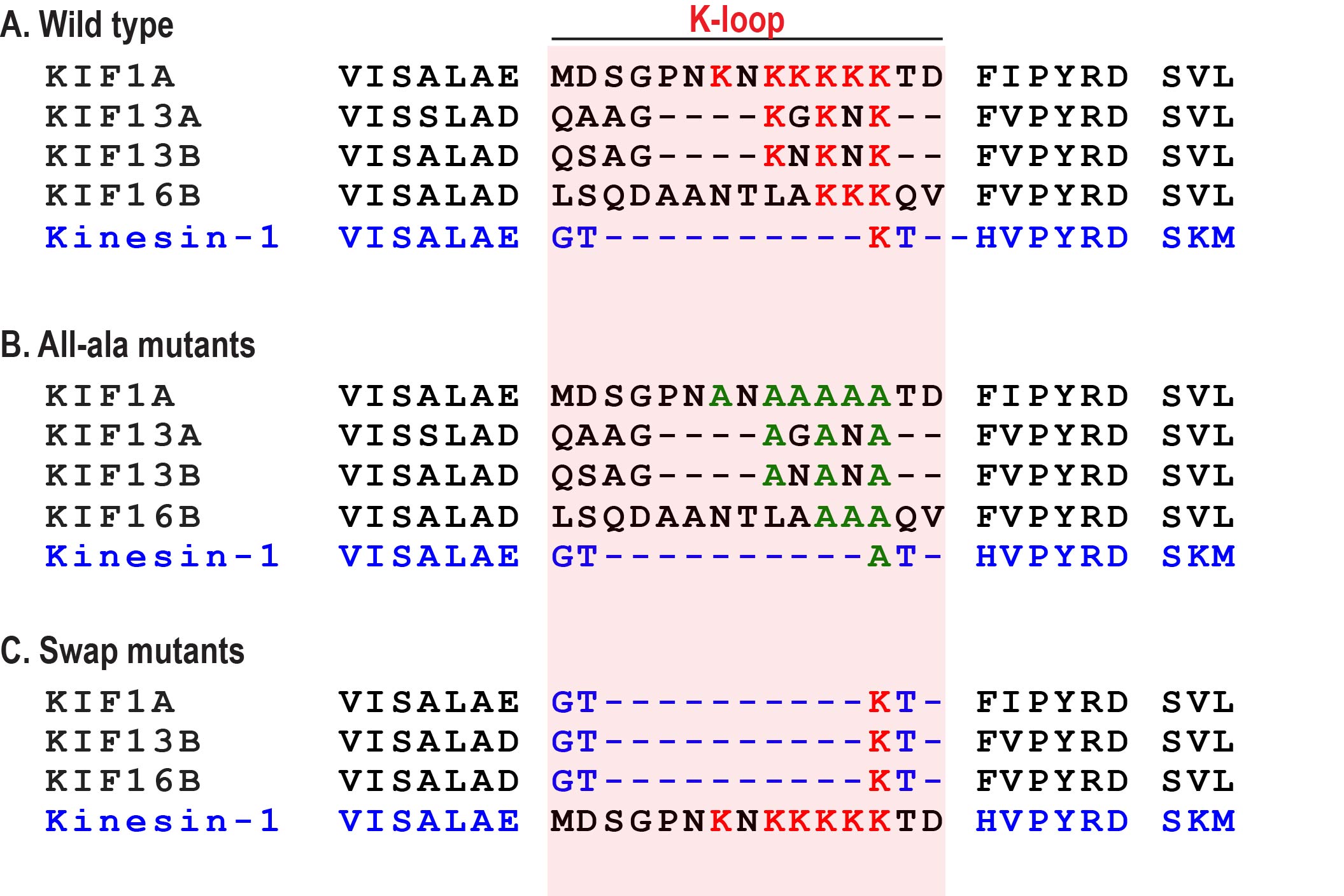
**

**Fig. S5: Amino acid sequence alignment of kinesin-3 and kinesin-1 K-loop and their mutants.** (A) Wild-type kinesin-3 (black text) and kinesin-1 (blue text). (B) All-Alanine mutants, in which all the positively charged lysine residues are mutated to alanine in the kinesin-3 and kinesin-1 K-loop regions. (C) Swap mutants, replacement of kinesin-3 K-loop with that of kinesin‑1 and the replacement of kinesin-1 K-loop with that of KIF1A.

**
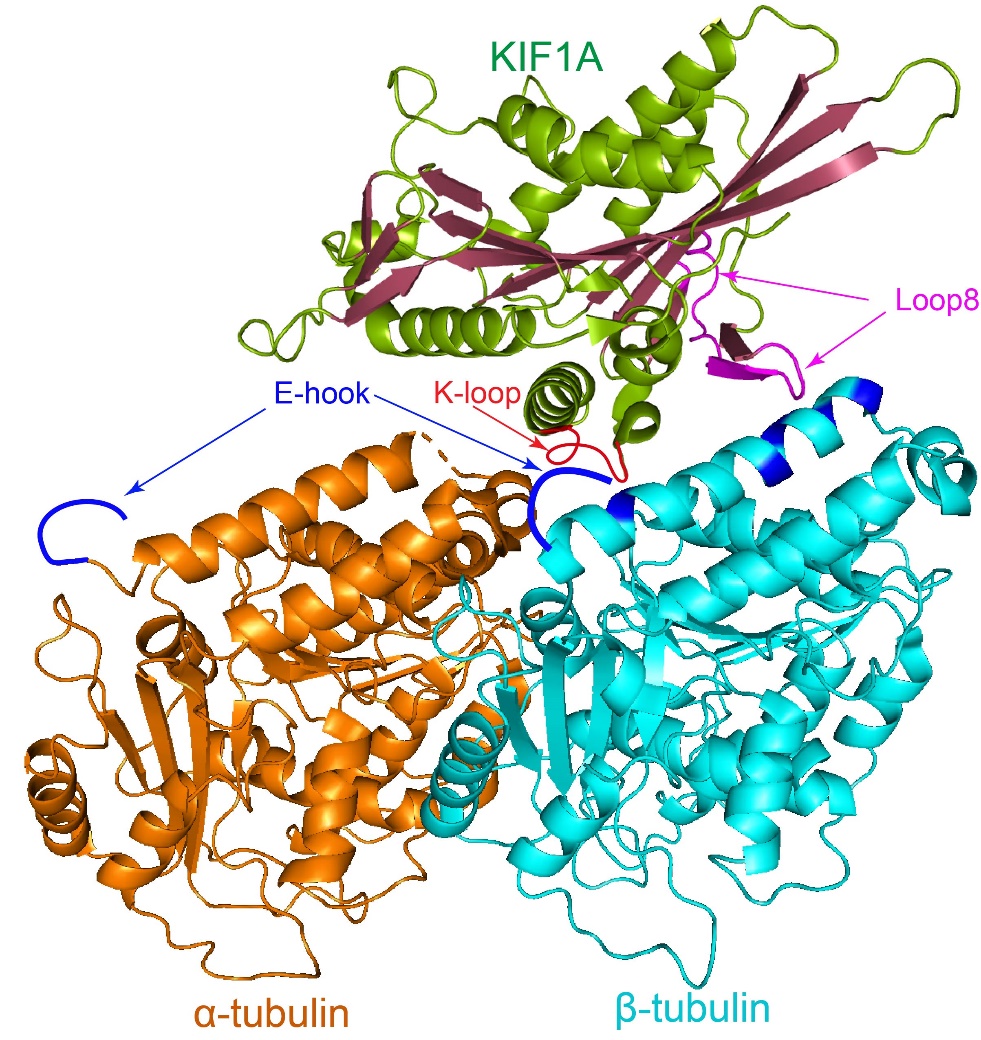
**

**Fig. S6: Ribbon diagram of KIF1A motor domain interacting with tubulin subunits.** Cartoon diagram showing the position of the conserved K-loop (red) and loop8 (magenta) of KIF1A (PDB 2HXF) interacting with the negatively charged residues in the E-hooks (blue) at the C-terminal tail regions of tubulin dimers.

**
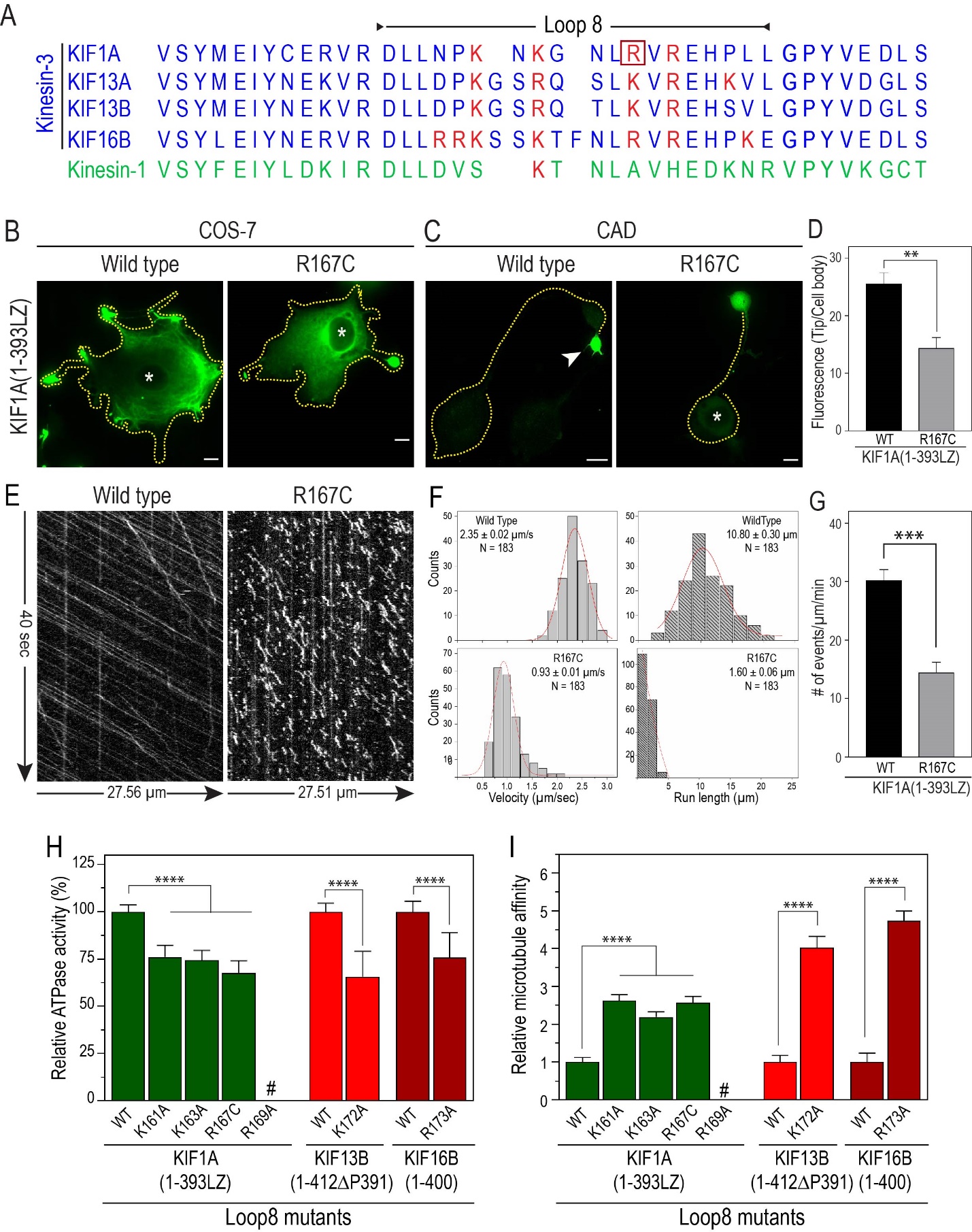
**

**Fig. S7. Loop8 contributes to the strong microtubule-binding affinity for kinesin-3 motors.** (A) Amino acid sequence alignment of the loop8 region of mammalian kinesin-3 family motors (blue text) and kinesin-1 (green text). Clusters of positively charged residues (red text). COS-7 (B) and CAD (C) cells expressing KIF1A(1-393LZ) wild-type and a loop8 mutant (R167C). (D) Quantification of motor accumulation at the neurite tip. The mean ± SEM for wild-type and mutant motors were plotted as the ratio of average fluorescence intensity in the neurite tip to that in the cell body. (E-F) Single-molecule motility assays of KIF1A(1-393LZ) wild-type and R167C mutant motors. The C-terminal 3xmCit tagged wild type and mutant motors were expressed in COS-7 cells and their lysates were used to measure the single-molecule motility properties. (E) Representative kymographs of wild-type or mutant single kinesin motors processively walking (white diagonal lines) along the microtubule. Distance is on x-axis (horizontal arrow) and time is on y-axis (vertical arrow). (F) Velocity (left panel) and run length (right panel) histograms of KIF1A(1-393LZ) wild type and R167C mutant motors fit to a single Gaussian. Average velocity and run length of the corresponding population of motors (N) are indicated on top-right or left-corner as mean ± SEM. Data presented from three independent experiments. (G) Quantification of the MT landing rates for the indicated wild-type and a loop 8 mutant (R167C). Yellow dotted line indicates the cell boundary, arrowhead indicates neurite tip and asterisk indicates the nucleus. Scale bars, 10 μm. Statistical difference calculated using Student’s *t*-test (***p<0.0005). (H-I) Relative ATP hydrolysis rate (H) and microtubule affinity (I) of wild type and kinesin-3 loop8 mutants. **#**, could not measure the ATPase activity due to protein expression problems. The ATPase activity of wild-type motor is considered as 100% and the relative activity of the loop8 mutants was then calculated. Values from three independent experiments.

**
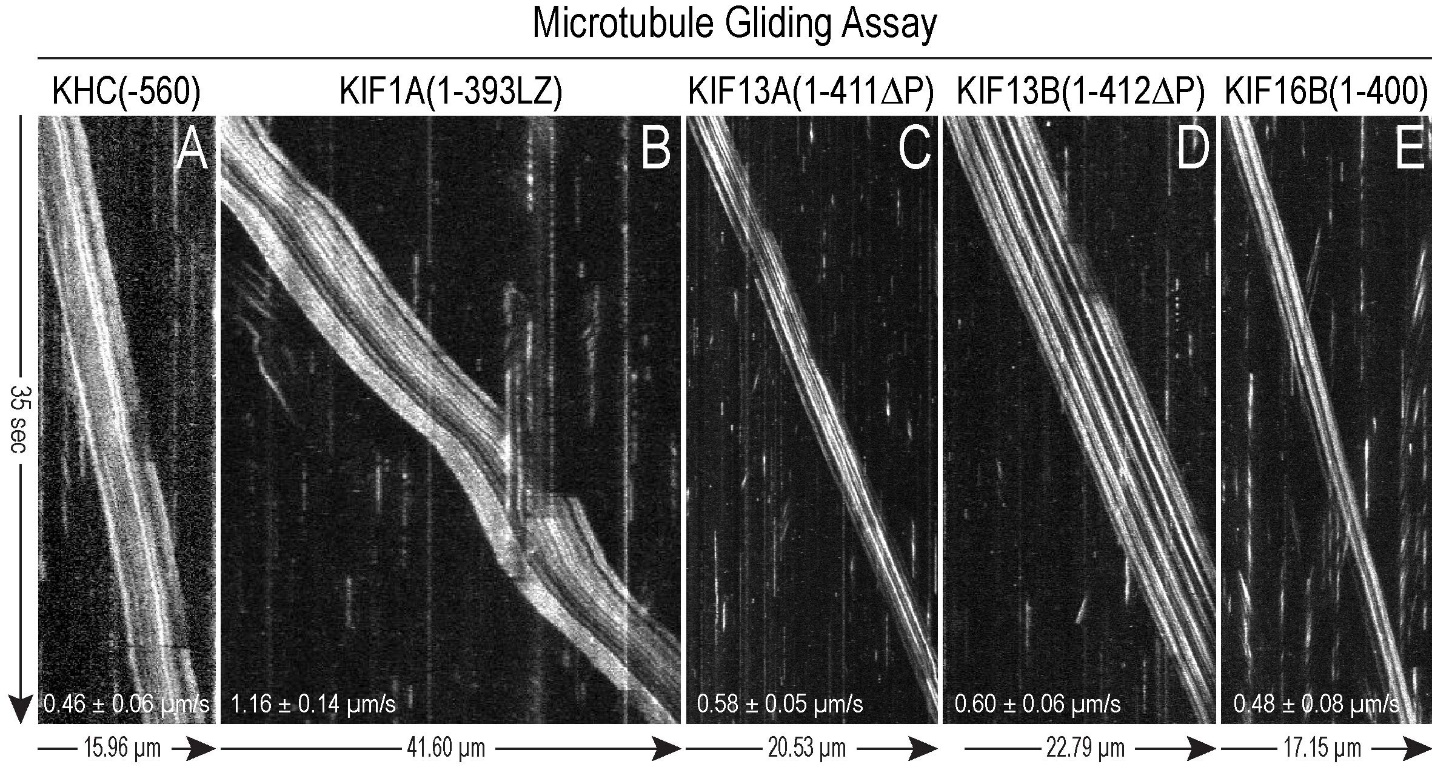
**

**Fig. S8. Multi-motor microtubule gliding analysis of kinesin-3 motors.** (A-E) Representative kymographs show MT gliding driven by the indicated motors (A) KHC (1-560), (B) KIF1A(1-393LZ), (C) KIF13A(1-411ΔP), (D) KIF13B(1-412ΔP) and (E) KIF16B(1-400) moving with uniform velocity. Time on the y-axis and distance on the x-axis. MT gliding velocities are described as mean ± SEM on bottom-left corner.

**
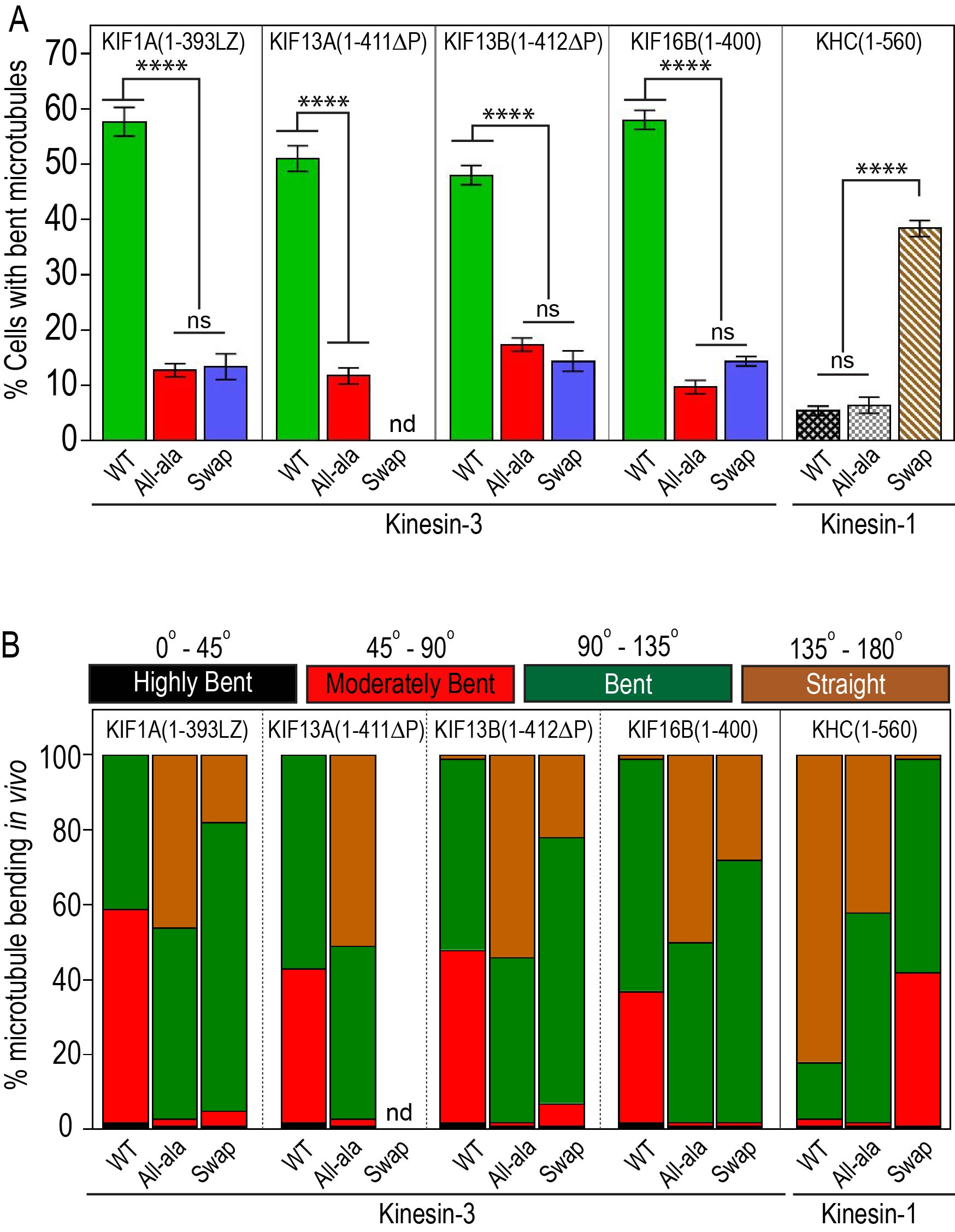
**

**Fig. S9.** **Kinesin-3 motors influence microtubule bending *in vivo*.** (A) Quantification of cotransfected cells exhibiting curved MTs for wild-type or K-loop mutants; alanine (All-ala) or Swap (K-loop swap) mutants. n = 110–130 cells each. Statistical difference calculated using Student’s t-test (****p<0.0001), ns (non-significant), The values and error bars represent mean ± SEM (B) Quantification percent cotransfected cells exhibiting microtubule-bending angle for wild-type or K-loop mutants; alanine (All-ala) or Swap (K-loop swap) mutants. n = 30-35 cells each.. nd (not determined). Values from three independent experiments.

**Movie legends**

**Movie S1. Microtubule gliding assay of constitutively active kinesin-1 motor, KHC(1-560).** TIRF imaging of MT gliding by KHC(1-560), kinesin-1 motor purified from Sf9-baculovirus system. Movie was acquired at 100 msec exposure and shown at 60 frames/sec. Scale bar, 10 μm. Selected bending events are marked by arrow.

**Movie S2. Microtubule gliding assay of constitutively active kinesin-3 motor, KIF1A(1-393LZ).** TIRF imaging of MT gliding by KIF1A(1-393LZ), kinesin-3 motor purified from Sf9-baculovirus system. Movie was acquired at 100 msec exposure and shown at 60 frames/sec. Scale bar, 10 μm. Selected bending events are marked by arrow.

**Movie S3. Microtubule gliding assay of constitutively active kinesin-3 motor, KIF13A(1-411ΔP).** TIRF imaging of MT by KIF13A(1-411ΔP), kinesin-3 motor purified from Sf9-baculovirus system. Movie was acquired at 100 msec exposure and shown at 60 frames/sec. Scale bar, 10 μm. Selected bending events are marked by arrow.

**Movie S4. Microtubule gliding assay of constitutively active kinesin-3 motor, KIF13B(1-412ΔP).** TIRF imaging of MT gliding by KIF13B(1-412ΔP), kinesin-3 motor purified from Sf9-baculovirus system. Movie was acquired at 100 msec exposure and shown at 60 frames/sec. Scale bar, 10 μm. Selected bending events are marked by arrow.

**Movie S5. Microtubule gliding assay of constitutively active kinesin-3 motor, KIF16B(1-400).** TIRF imaging of MT gliding by KIF16B(1-400), kinesin-3 motor purified from Sf9-baculovirus system. Movie was acquired at 100 msec exposure and shown at 60 frames/sec. Scale bar, 10 μm. Selected bending events are marked by arrow.

**Movie S6. Microtubule gliding assay of KIF1A(1-393LZ) All-alanine mutant.** TIRF imaging of MT gliding by KIF1A(1-393LZ) All-alanine mutant, in which the lysine residues in the K-loop were mutated to alanine, purified from Sf9-baculovirus system. Movie was acquired at 100 msec exposure and shown at 60 frames/sec. Scale bar, 10 μm. Selected bending events are marked by arrow.

**Movie S7. Microtubule gliding assay of KIF1A(1-393LZ) Swap mutant.** TIRF imaging of MT gliding by KIF1A(1-393LZ) Swap mutant, in which the K-loop of KIF1A motor is swapped with that of kinesin-1, purified from Sf9-baculovirus system. Movie was acquired at 100 msec exposure and shown at 60 frames/sec. Scale bar, 10 μm. Selected bending events are marked by arrow.

**Movie S8. Microtubule gliding assay of KHC(1-560) Swap mutant.** TIRF imaging of MT gliding by KHC(1-560) Swap mutant, in which the K-loop of kinesin-1 motor is swapped with that of KIF1A, purified from Sf9-baculovirus system. Movie was acquired at 100 msec exposure and shown at 60 frames/sec. Scale bar, 10 μm. Selected bending events are marked by arrow.
