# Supplementary material for: Kinesin-3 motors are fine-tuned at the molecular level to endow distinct mechanical outputs
Source: BMC Biol. 2022 Aug 10;20:177. doi: 10.1186/s12915-022-01370-8 (PMC9364601; doi:10.1186/s12915-022-01370-8)
Supplement: Supplementary file 2 — Additional file 2: Table S1. Summary of ATPase properties of full-length kinesin-1 and kinesin-3 motors. [file 12915_2022_1370_MOESM2_ESM.docx]

**Table S1: ATPase analysis of full-length kinesin-1 and kinesin-3 motors.**

| **Motor** | **ATPase activity (s^-1^)** | **Microtubule concentration (µM)** |
| --- | --- | --- |
| KHC | 15.98 | 5.0 |
| KIF1A | 48.02 | 20.0 |
| KIF13A | 30.05 | 8.0 |
| KIF13B | 25.00 | 8.0 |
| KIF16B | 35.00 | 8.0 |
