# Supplementary figures and images for: Kinesin-3 motors are fine-tuned at the molecular level to endow distinct mechanical outputs
Source: BMC Biol. 2022 Aug 10;20:177. doi: 10.1186/s12915-022-01370-8 (PMC9364601; doi:10.1186/s12915-022-01370-8)

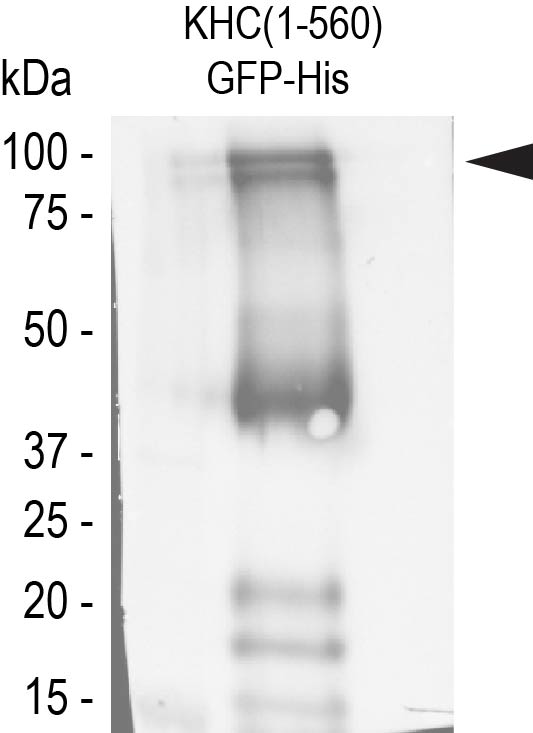

Supplement: Supplementary file 4 — Additional file 4. Uncropped blot for Fig. S1B. [file 12915_2022_1370_MOESM4_ESM.jpg]

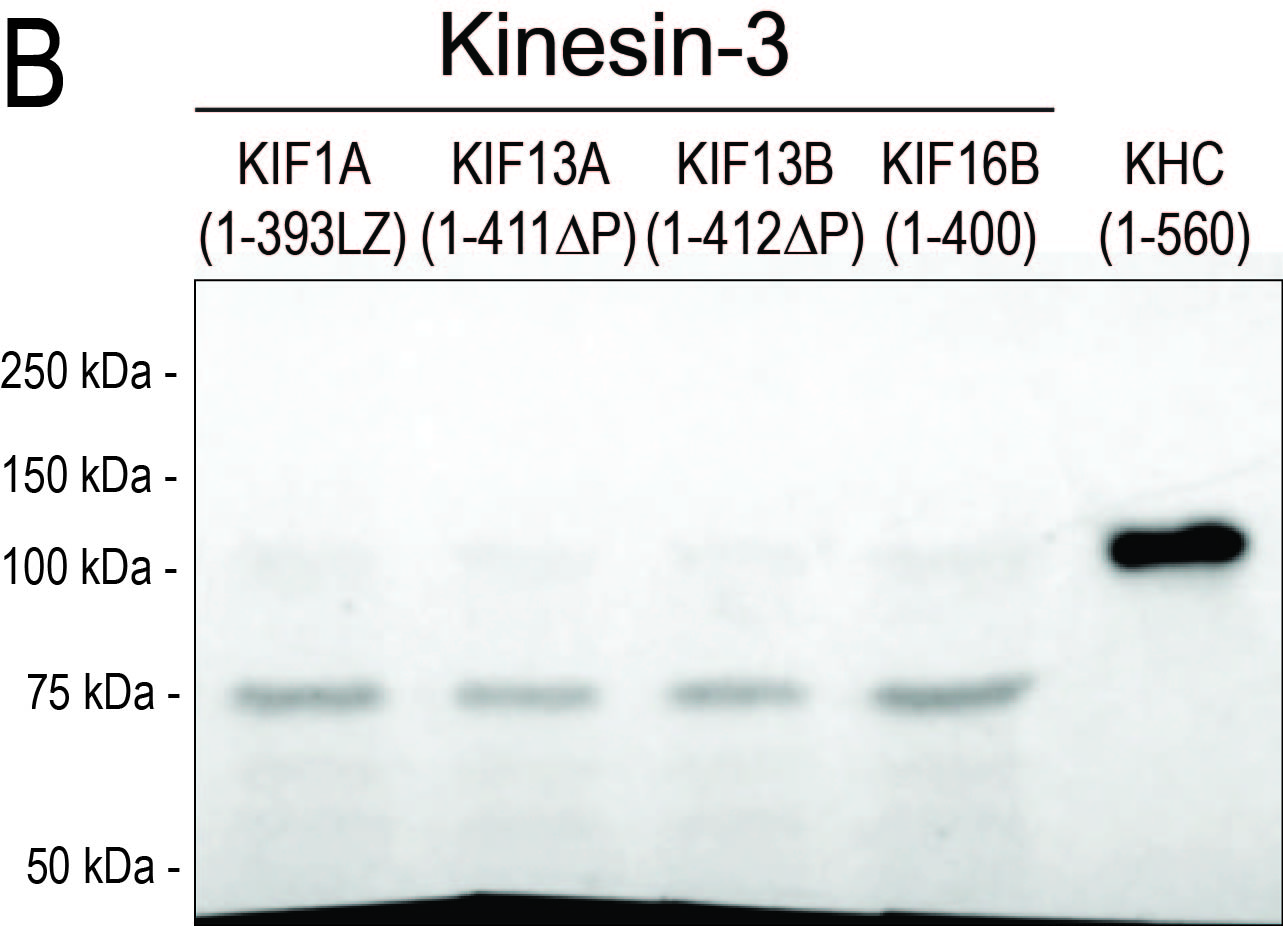

Supplement: Supplementary file 5 — Additional file 5. Uncropped blot for Fig. S3B. [file 12915_2022_1370_MOESM5_ESM.jpg]

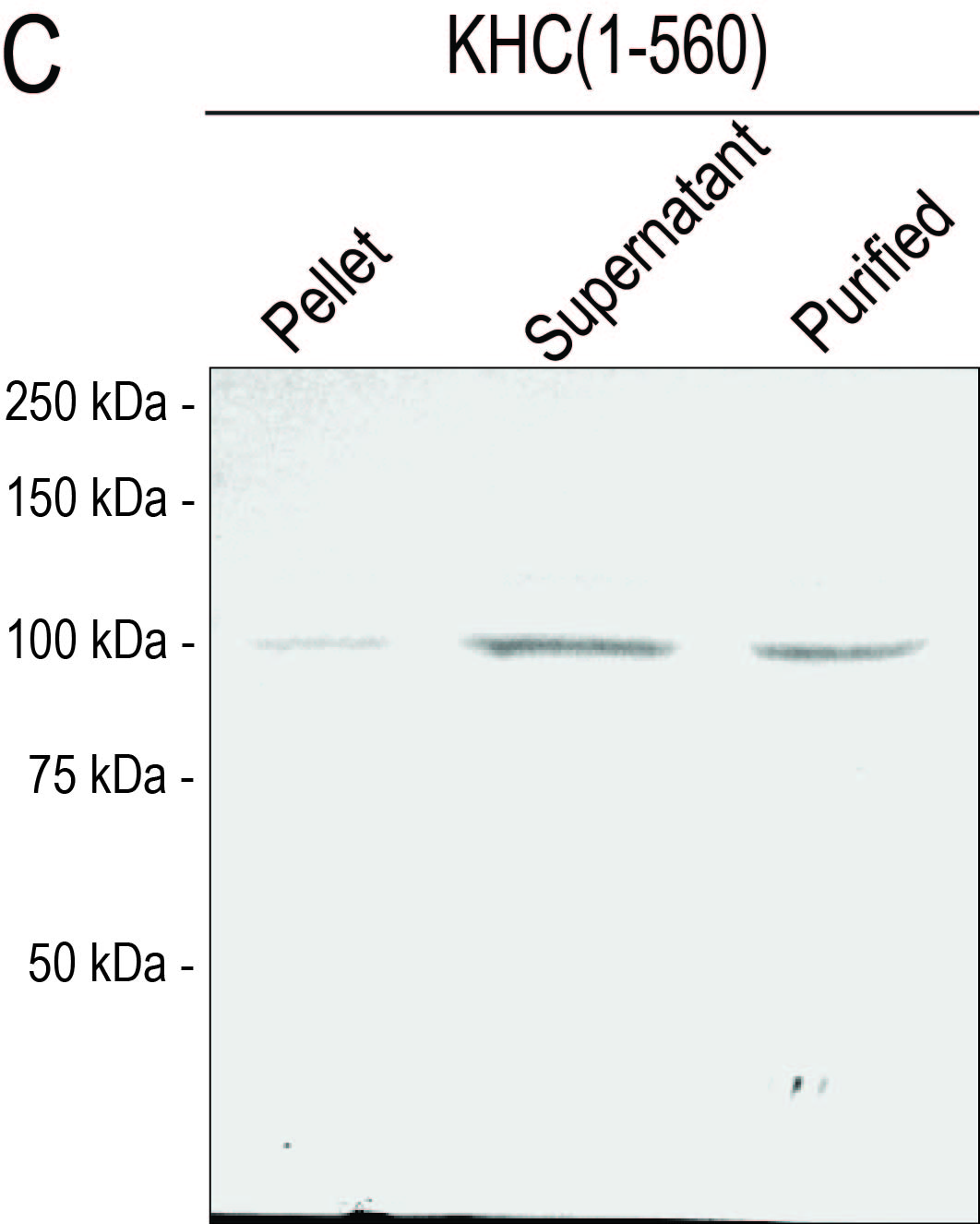

Supplement: Supplementary file 6 — Additional file 6. Uncropped blot for Fig. S3C. [file 12915_2022_1370_MOESM6_ESM.jpg]
